# Iron overload disorders in adults: a comprehensive review of gonadal function, reproductive, and sexual health

**DOI:** 10.1093/humupd/dmag010

**Published:** 2026-05-08

**Authors:** Francesco Carlomagno, Marta Tenuta, Andrea Sansone, Giulia Rastrelli, Francesca Sciarra, Biagio Cangiano, Andrea M Isidori, Daniele Gianfrilli, Csilla Krausz

**Affiliations:** Department of Experimental Medicine, Sapienza University of Rome, Rome, Italy; Department of Experimental Medicine, Sapienza University of Rome, Rome, Italy; Section of Endocrinology and Medical Sexology, Department of Systems Medicine, University of Rome Tor Vergata, Rome, Italy; Department of Experimental and Clinical Biomedical Sciences ‘Mario Serio’, University of Florence, Florence, Italy; Andrology and Gender Endocrinology Unit, Careggi Hospital, Florence, Italy; Department of Experimental Medicine, Sapienza University of Rome, Rome, Italy; Department of Medical Biotechnology and Translational Medicine, University of Milan, Milan, Italy; Department of Endocrine and Metabolic Diseases, IRCCS Istituto Auxologico Italiano, Milan, Italy; Department of Experimental Medicine, Sapienza University of Rome, Rome, Italy; Department of Experimental Medicine, Sapienza University of Rome, Rome, Italy; Department of Experimental and Clinical Biomedical Sciences ‘Mario Serio’, University of Florence, Florence, Italy; Andrology and Gender Endocrinology Unit, Careggi Hospital, Florence, Italy

**Keywords:** iron overload, reproductive dysfunction, hypogonadism, fertility, pregnancy, sexual function, ferroptosis, iron chelation therapy, thalassaemia, haemochromatosis

## Abstract

**BACKGROUND:**

Iron overload (IO) disorders, including thalassaemias, hereditary haemochromatosis, and transfusion-dependent anaemias, represent a growing clinical challenge with widespread systemic implications. Reproductive dysfunction remains severely underappreciated despite its high prevalence. Hormonal changes due to iron toxicity are frequently reported, yet are seldom the focus of reproductive medicine, causing fragmented knowledge, inconsistent clinical approaches, and a lack of consensus guidelines.

**OBJECTIVE AND RATIONALE:**

This review synthesizes evidence on the impact of IO on male and female reproductive function, including gonadal dysfunction, impaired fertility, sexual dysfunction, and endocrine-metabolic complications. By addressing gaps in study design, diagnostic criteria, and management, we aim to provide the first comprehensive, expert-driven synthesis on the topic, integrating clinical, translational, and mechanistic insights to establish a structured framework for future research and patient care.

**SEARCH METHODS:**

A systematic literature search was conducted across PubMed, Scopus, and Web of Science, including studies up to May 2025. Search terms included ‘iron overload’, ‘thalassemia’, ‘hemochromatosis’, ‘hypogonadism’, ‘fertility’, ‘spermatogenesis’, ‘ovarian insufficiency’, and ‘pregnancy’. Quantitative synthesis involved pooling data on prevalence rates of hypogonadism, semen abnormalities, primary and secondary amenorrhoea, age at menarche, and pregnancy outcomes.

**OUTCOMES:**

Gonadal dysfunction primarily arises from iron deposition within the hypothalamic–pituitary–gonadal axis, coupled with oxidative damage to Leydig and Sertoli cells in males, disrupting testosterone synthesis and spermatogenesis, and to ovarian follicles and granulosa cells in females, causing reduced ovarian reserve and altered hormonal signalling. Iron-induced hypogonadism is the most frequent endocrine complication, significantly impacting reproductive health and quality of life. Our analysis of 1201 men and 2134 women indicated hypogonadism, reflecting impaired testicular endocrine function, in 47.0% of men; among those specifically assessed for spermatogenesis, over half presented azoospermia (17.6%) or other sperm abnormalities (37.5%). In women, primary amenorrhoea was reported in 45.7%, secondary amenorrhoea in 20.0%, and the weighted mean age at menarche was delayed (14.4 ± 2.1 years). Sexual dysfunction, notably erectile dysfunction, commonly accompanies hypogonadism, further impairing quality of life. Female sexual health has not been investigated at all. Pregnancy is increasingly achievable, but remains clinically challenging. Across 3536 reviewed pregnancies, ART was required in ∼20%, miscarriage occurred in 11.2%, and caesarean section was used in ∼80%. Mean gestational age at delivery was 37.1 ± 3.1 weeks, and mean birth weight was 2.64 ± 0.68 kg. Besides gonadal damage (direct or pituitary-related), systemic iron-related endocrine and metabolic disturbances, including hypothyroidism, growth hormone deficiency, diabetes mellitus, and cardiovascular disease, further aggravate reproductive impairments. Although effective iron chelation therapy reduces the systemic iron burden and is effective in preventing endocrine complications when initiated early, evidence supporting the reversal of established reproductive dysfunction remains limited, highlighting the need to optimize iron control from a young age to preserve reproductive health.

**WIDER IMPLICATIONS:**

This review underscores the critical need for standardized gonadal screening to facilitate personalized reproductive care and early intervention in subjects with IO disorders. We propose an integrated clinical framework, combining early endocrine monitoring, fertility preservation protocols, and reproductive counselling. Future multidisciplinary research should prioritize prospective studies with clearly defined reproductive endpoints and explore optimized chelation strategies to safeguard reproductive potential. Addressing these gaps will fundamentally reshape clinical management, bridging haematology, endocrinology, and reproductive medicine.

## Graphical abstract

**Graphical Abstract:**
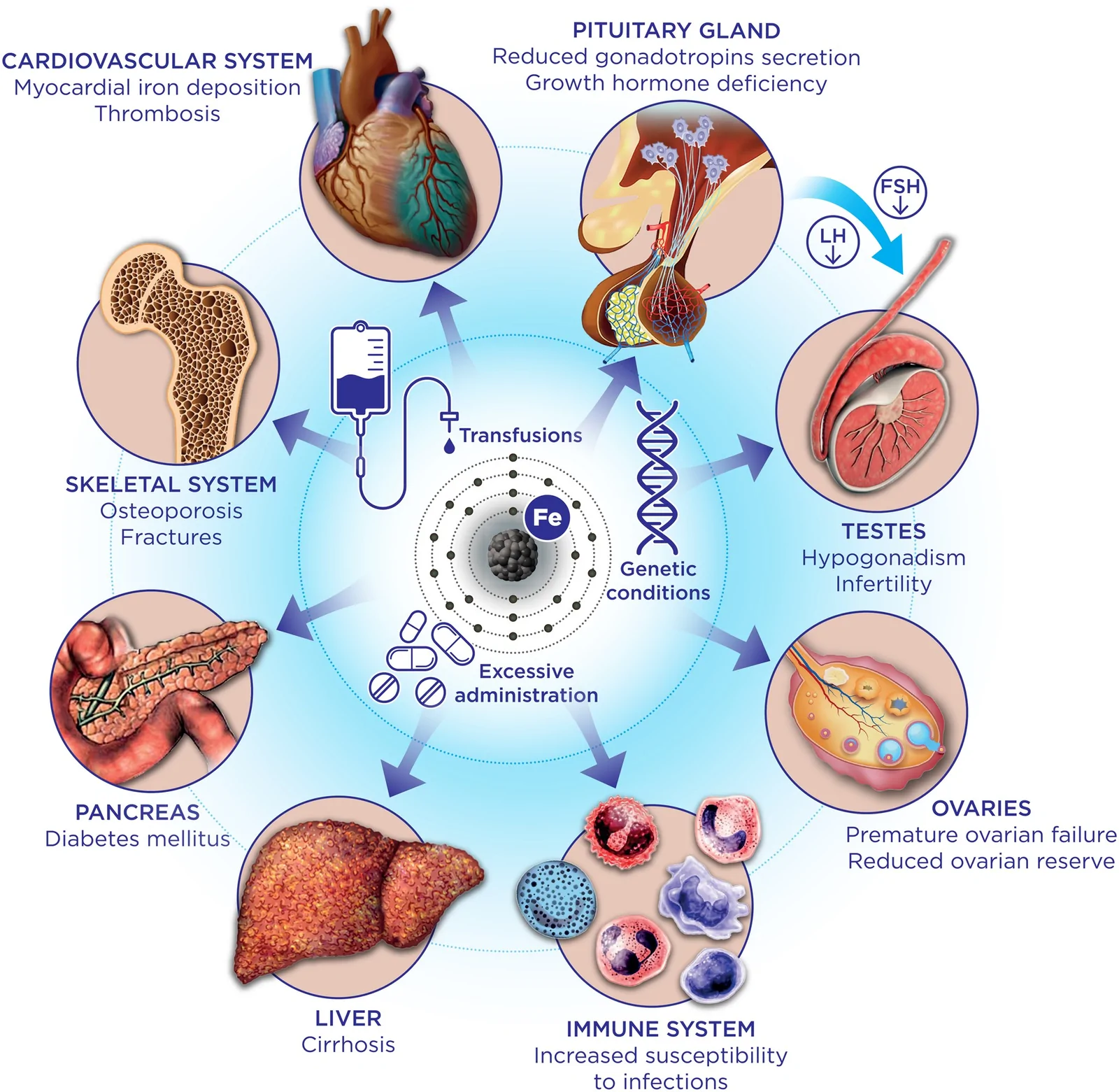
**Systemic iron overload from genetic or transfusional causes damages endocrine and reproductive organs via tissue iron deposition and ferroptosis, driving hypogonadism, infertility, and multi-system complications**.

## Introduction

Iron is an essential micronutrient required for a wide range of biological processes, from oxygen transport and energy production to DNA synthesis. However, when its homeostasis is disrupted, iron accumulates in tissues, leading to iron overload (IO), a condition characterized by excessive iron deposition and progressive organ damage.

IO can result from genetic mutations (Table 1) or acquired causes. Primary or hereditary haemochromatosis (HH) is linked to mutations in genes regulating iron absorption and storage, including *HFE*, *HAMP*, and *TFR2*, which disrupt the delicate balance of iron metabolism (Pietrangelo, 2010). Secondary haemochromatosis, often due to recurrent blood transfusions or excessive iron supplementation, is frequently observed in conditions like β-thalassaemia major (β-TM), transfusion-dependent β-thalassaemia intermedia (β-TI), and sickle cell anaemia (SCA). Throughout this review, the term IO is used to describe the pathological accumulation of iron, irrespective of its aetiology, whereas HH refers specifically to genetically determined forms of IO and encompasses distinct subtypes based on the underlying gene mutation.

**Table 1.** Genetic factors involved in iron overload.

Condition	Gene	Transmission	Mechanism of overload
**Primary haemochromatosis**			
Type 1 haemochromatosis	*HFE*	AR	Excessive iron absorption
Type 2A haemochromatosis	*HJV (haemojuvelin)*	AR	Excessive iron absorption
Type 2B haemochromatosis	*HAMP (hepcidin)*	AR	Excessive iron absorption
Type 3 haemochromatosis	*TFR2 (transferrin receptor-2)*	AR	Excessive iron absorption
Type 4 haemochromatosis	*FPN1 (ferroportin)*	AD	Excessive iron absorption
**Secondary haemochromatosis**			
α-Thalassaemia	*HBA1*, *HBA2*	AR	Repeated blood transfusions
β-Thalassaemia	*HBB*	AR	Repeated blood transfusions
Chronic haemolytic anaemia	*ANK1*, *SPTA1*, *SPTB*, *SLC4A1*, *EPB42 (spherocytosis)* *G6PD (G6PD deficit)*	AD or AR XL (G6PD deficiency)	Repeated blood transfusions
Congenital sideroblastic anaemia	*ALAS2*, *SLC25A38*, *GLRX5*, *PUS1*, *ABCB7*, *HSPA9*	XL (ALAS2) AR or AD (other forms)	Repeated blood transfusions
Sickle cell anaemia	*HBB (Glu6Val mutation)*	AR	Repeated blood transfusions
Diamond–Blackfan anaemia	*RPS19*, *RPL5*, *RPL11*, *RPS26*, etc.	AD (often *de novo* mutations)	Repeated blood transfusions
Hereditary dyserythropoietic anaemia	*CDAN1*, *CDIN1*, *SEC23B*, *KIF23*	AR (CDAN1, CDIN1, SEC23B) AD (KIF23)	Repeated blood transfusions
Fanconi anaemia	*FANCA*, *FANCC*, *FANCG*, etc.	AD or AR	Repeated blood transfusions

AD, autosomal dominant; AR autosomal recessive; XL, X-linked.

With advances in iron chelation therapies (ICT), survival and quality of life for patients with IO disorders have markedly improved (Borgna-Pignatti *et al.*, 2004; Vogiatzi *et al.*, 2009; Derchi *et al.*, 2011). As a result, the extended lifespan of these individuals has brought attention to the long-term consequences of iron toxicity, particularly its effects on the hypothalamic–pituitary–gonadal (HPG) axis. Among these, hypogonadism is the most prevalent and has the most clinical impact, affecting sexual health, fertility, and systemic well-being (Borgna-Pignatti *et al.*, 2004; McDermott and Walsh, 2005; Toumba *et al.*, 2007; Pafumi *et al.*, 2011; Cappellini *et al.*, 2021; Spaziani *et al.*, 2023a).

This narrative review explores the prevalence, pathophysiology, and clinical implications of hypogonadism in adults with IO disorders. It also examines therapeutic strategies, including ICT, testosterone replacement therapy (TRT), and estrogen replacement therapy (ERT), and discusses factors influencing gonadal and sexual function, fertility, and pregnancy outcomes in affected men and women. A visual summary of the systemic consequences of IO, both endocrine and non-endocrine, is provided, highlighting the mechanisms contributing to iron accumulation and its impact on gonadal function in both sexes.

### Iron metabolism and ferroptosis

Iron is a mineral required for essential biological processes such as the Krebs cycle, oxygen transport, and DNA synthesis. In biological systems, it exists in two forms: ferrous (Fe^2+^) and ferric (Fe^3+^) iron. In the bloodstream, the insoluble ferric form is transported bound to proteins like transferrin, while the soluble ferrous form is actively used in intracellular processes.

Iron homeostasis is a finely tuned system balancing absorption, storage, and recycling (MacKenzie *et al.*, 2008; Hentze *et al.*, 2010). Dietary iron, mainly Fe^3+^, is reduced to Fe^2+^ by ascorbate ferrireductase in the intestine and absorbed via DMT1 (Shawki *et al.*, 2015). Once absorbed, Fe^2+^ is exported into the bloodstream by ferroportin and oxidized back to Fe^3+^ by ferroxidases, such as hephaestin and ceruloplasmin, before binding to transferrin for systemic distribution (Donovan *et al.*, 2005). Cells acquire iron mainly through transferrin receptor (TfR)-mediated endocytosis, although studies report a role for L-type calcium channels (LTCC) in conditions of IO, and preclinical experimental data suggest that pharmacological inhibition of LTCCs may mitigate iron accumulation in cardiomyocytes (Oudit *et al.*, 2006; Kumfu *et al.*, 2016). Within the cell, iron is either stored in the ferritin protein, preventing its participation in oxidative reactions, or it is utilized in metabolic pathways (Lucesoli and Fraga, 1995; Kramer *et al.*, 2012). Thus, once inside the cell, Fe^2+^ can be used by the cell, or it can be reduced again to Fe^3+^ to be stored by ferritin or secreted in the bloodstream. Serum ferritin therefore acts as a marker of body iron storage: its concentration increases in IO and decreases in iron deficiency. Hepcidin is the master regulator of iron homeostasis, modulating ferroportin to control iron release from enterocytes and macrophages. During iron deficiency or increased demand, hepcidin synthesis is suppressed to enhance absorption and mobilization. Conversely, in IO, hepcidin is upregulated, inhibiting ferroportin, thus reducing systemic iron levels (Knutson *et al.*, 2005).

IO leads to tissue damage primarily through ferroptosis, a form of iron-dependent cell death triggered by reactive oxygen species (ROS) generated via the Fenton reaction. At the molecular level, ferroptosis is driven by the convergence of IO, enhanced lipid peroxidation (notably via ACSL4-dependent phospholipid remodelling), and impaired antioxidant defences, primarily mediated by the System Xc^−^/glutathione/glutathione peroxidase 4 (GPX4) axis (Ma *et al.*, 2022; Zhang *et al.*, 2024). Iron-dependent enzymes, like arachidonate lipoxygenase, amplify lipid peroxidation, producing reactive aldehydes that impair protein function, and lead to inflammation (Tang and Kroemer, 2020). Altogether, these processes determine irreversible cellular injury, particularly in iron-rich organs such as the liver, heart, and endocrine glands (Musumeci *et al.*, 2014; Rossi *et al.*, 2016).

While most mechanistic insights derive from experimental systems, emerging data in human tissues support the biological role of ferroptosis in reproductive pathology. In human testicular tissue, early proteomic and immunohistochemical studies have documented altered expression of *GPX4*, a central inhibitor of ferroptosis, in patients with spermatogenic failure (Huo *et al.*, 2008). More recently, transcriptomic, ultrastructural, and biochemical analyses of testicular biopsies from men with idiopathic non-obstructive azoospermia have demonstrated downregulation of ferroptosis-related genes (including *GPX4*), iron accumulation, increased lipid peroxidation, and mitochondrial morphological features consistent with ferroptotic cell death (Liao *et al.*, 2023). In addition, studies in infertile men have reported elevated ACSL4 levels, correlations between ferritin, isoprostanes, and impaired semen parameters, and localization of lipid peroxidation-promoting enzymes in spermatozoa, collectively indicating activation of ferroptosis-related pathways in human germ cells (Moretti *et al.*, 2024).

Evidence of ferroptosis is also emerging in female reproductive tissues. Clinical studies in women with reproductive-metabolic disorders, such as polycystic ovary syndrome (PCOS), have shown dysregulated systemic iron metabolism and, importantly, upregulation of ferroptosis-associated genes (e.g. *ACSL4*, *GPX4*, *SLC7A11*, *FTH1*) in human placental tissue, linking iron-dependent lipid peroxidation to adverse reproductive and pregnancy outcomes (Wang *et al.* 2025). The PCOS model has also provided indirect insights into the effect of ferroptosis on ovarian function. Indeed, iron excess has been implicated in insulin resistance and metabolic dysfunction in PCOS, especially in women with impaired glucose tolerance, with a bidirectional interaction whereby iron influences insulin secretion and sensitivity, while insulin modulates iron metabolism (Fernandez-Real *et al.*, 2002; Swaminathan *et al.*, 2007; Escobar-Morreale, 2012).

The mechanistic evidence derived from *in vitro* models of human granulosa cells from women with PCOS has suggested iron-induced ferroptosis as a potential contributor to ovarian dysfunction. Indeed, in granulosa cells from PCOS patients, elevated intracellular Fe^2+^ and increased lipid peroxidation, together with downregulation of *GPX4* and *FTH1* and upregulation of *NCOA4*, promoting ferritin degradation and intracellular iron release, have been reported, supporting a ferroptosis-related mechanism of cellular injury (Li *et al.*, 2024). Although the degree of iron excess in PCOS is significantly milder than in haematological IO disorders, iron-mediated metabolic disturbances may contribute to ovarian dysfunction and menstrual irregularities in this setting.

While these findings cannot be directly extrapolated to women with systemic IO disorders, they provide biological plausibility for a role of iron-mediated oxidative and metabolic stress in female reproductive and sexual health.

### Pituitary gland

Chronic IO disrupts the HPG axis, ultimately leading to hypogonadism and infertility. Iron deposition occurs across all pituitary cell types, and the anterior pituitary is highly sensitive to iron-induced damage (De Sanctis *et al.*, 2013; Roussou *et al.*, 2013). Gonadotrophin-secreting cells are particularly vulnerable (Kontogeorgos *et al.*, 1996), resulting in reduced responsiveness to GnRH (Roussou *et al.*, 2013), which leads to decreased sex hormone production and gamete formation (De Sanctis *et al.*, 2004; Toumba *et al.*, 2007), as clinical consequences of hypogonadotropic hypogonadism (Lucesoli and Fraga, 1999; De Sanctis *et al.*, 2013; Roussou *et al.*, 2013). Furthermore, reduced FSH levels may also have extra-gonadal effects, for instance, on bone metabolism and the immune system (Spaziani *et al.*, 2023b).

While gonadotrophs are particularly affected, IO can also damage other pituitary cell types, contributing to broader endocrine dysfunctions, including hypothalamic–pituitary–adrenal axis impairment and reduced GH/insulin-like growth factor-1 (IGF-1) levels (Uitz *et al.*, 2013). Indeed, somatotroph cells can be affected by IO, with a reported prevalence of growth hormone deficiency (GHD) of up to ∼50% (Grundy *et al.*, 1994; Aydinok *et al.*, 2002; Dhouib *et al.*, 2018). The coexistence of GHD in this population is particularly relevant, as it may negatively affect the HPG axis, as supported by a growing body of evidence (Tenuta *et al.*, 2020).

Pituitary MRI is sensitive to iron due to its superparamagnetic properties and can detect pituitary iron deposition through specific sequences (T2, T2*, R2* mapping) (St Pierre *et al.* 2005). MRI signal abnormalities may represent early markers of iron deposition, preceding measurable volume loss, which often becomes evident in advanced disease stages (Berkovitch *et al.*, 2000; Noetzli *et al.*, 2012a). Hypogonadal patients with thalassaemia show lower pituitary height, volume, and T2 relaxation times compared to healthy controls (Argyropoulou *et al.*, 2001; Christoforidis *et al.*, 2007; Noetzli *et al.*, 2012b; Bozdağ *et al.*, 2018). Furthermore, T2/T2* hypointensity and pituitary volume are both associated with the degree of endocrine dysfunction (Berkovitch *et al.*, 2000; Hekmatnia *et al.*, 2010; Noetzli *et al.*, 2012b).

### Testicles

Iron is essential for male germ cell development, serving as a cofactor for DNA synthesis and cell growth during meiosis (Kaplan, 2002; Aitken and Curry, 2011). Although the precise mechanism of iron transport across the blood–testis barrier remains unclear, it is suggested that Sertoli cells play a pivotal role, importing iron via transferrin and its receptor, as demonstrated in animal models (Skinner and Griswold, 1982; Huggenvik *et al.*, 1984; Sylvester and Griswold, 1984; Gelly *et al.*, 1994).

Key proteins involved in iron metabolism, such as transferrin (and its receptor), DMT1, and ferroportin, have been detected in testicular tissues, alongside cytosolic and mitochondrial ferritin in Sertoli, germ, and Leydig cells, supporting the existence of an autonomous iron cell cycle within seminiferous tubules (Breucker *et al.*, 1985; Santambrogio *et al.*, 2007; Wolff *et al.*, 2011; Leichtmann-Bardoogo *et al.*, 2012). Ferritin concentrations are the highest in Sertoli cells, peritubular myoid cells, and early spermatocytes, with levels decreasing during gamete maturation (Leichtmann-Bardoogo *et al.*, 2012). Sertoli cells actively secrete transferrin and ferritin in seminal plasma under FSH and testosterone stimulation, underscoring the role of iron in spermatogenesis (Holmes *et al.*, 1982; Gabrielsen *et al.*, 2018).

In conditions of overload, iron deposition occurs particularly around seminiferous tubules, rather than within them, due to the effects of the blood–testis barrier, which effectively detaches iron homeostasis from the periphery, protecting germ cells from fluctuations in circulating iron levels (Leichtmann-Bardoogo *et al.*, 2012).

Iron-induced oxidative stress and chronic inflammation damage spermatids and spermatozoa (Aitken and Curry, 2011), as well as Leydig cells, ultimately impairing testosterone production (Marin *et al.*, 2010; Zhang *et al.*, 2011; Isidori *et al.*, 2014; Tremellen *et al.*, 2018; Bianchi, 2019; Sciarra *et al.*, 2023). As recently shown, germ cells exposed to IO undergo ferroptotic cell death through a cascade involving glutathione depletion, accumulation of lipid ROS, and downregulation of anti-ferroptotic markers (Moretti *et al.*, 2024), independently of classical apoptotic or necrotic mechanisms (Sun *et al.*, 2023). Iron accumulation also leads to mitochondrial and endoplasmic reticulum dysfunctions in germ cells and somatic cells, impairing spermatogenesis and steroidogenesis (Pantopoulos *et al.*, 2012; Kurniawan *et al.*, 2019; Yang *et al.*, 2022). Indeed, altered iron efflux has been linked to downregulation of key steroidogenic enzymes, including *StAR*, *CYP11A*, and *17β-HSD*, while endoplasmic reticulum stress promotes Leydig cell apoptosis, exacerbating androgen deficiency (Aigner *et al.*, 2014; Kurniawan *et al.*, 2019; Weiss *et al.*, 2019).

### Ovaries and uterus

Pathological iron accumulation has been documented in both ovaries and the uterus in IO. Similar to the testis, ROS can impair ovarian function by disrupting steroidogenesis and folliculogenesis, accelerating ovarian aging, and reducing ovarian volume and reserve (Tatone *et al.*, 2008; Asano, 2012; Rossi *et al.*, 2016; Hoque *et al.*, 2021). In animal models, iron has been shown to interfere with 17β-estradiol (E2) binding to estrogen receptors (ERα and ERβ), reducing E2-mediated effects (Murata *et al.*, 2014). *In vitro* studies further demonstrated that Fe^2+^ (but not Fe^3+^) inhibits granulosa cell proliferation by inducing cell cycle arrest at the G2/M phase through the ROS-mediated activation of the p38 MAPK/p53/p21 pathway (Chen *et al.*, 2017). This cascade leads to impaired granulosa cell function, decreased antral follicle count (AFC) and anti-Müllerian hormone (AMH) levels, and limits corpora lutea formation, while increasing follicular atresia (el-Deiry *et al.*, 1993; Xiong *et al.*, 1993; Appasamy *et al.*, 2008; Tatone *et al.*, 2008; Bajoria and Chatterjee, 2011; Asano, 2012; Hoque *et al.*, 2021).

An inverse relationship has been shown between non-transferrin-bound iron and AMH levels in β-TM (Singer *et al.*, 2011). For example, in a case–control study (of n = 43), AMH levels, ovarian volume, and AFC were significantly lower in women affected by β-TM than in age-matched healthy controls (Uysal *et al.*, 2017). Similar findings have been reported in transfusion-dependent thalassaemias (TDT) and SCA, highlighting a consistent compromise of ovarian reserve (Chang *et al.*, 2011; Kopeika *et al.*, 2019; Mensi *et al.*, 2019).

Iron deposition also affects the endometrium, as endometrial stromal cells express TfR1 and ferroportin, facilitating sustained iron uptake (Mori *et al.*, 2015). In a case series of patients with β-TM undergoing endometrial biopsy for infertility, glandular haemosiderosis was observed, particularly in the apical epithelium. Notably, iron deposition occurred in women with high serum ferritin in the range of 3.500–7.000 µg/l, despite undergoing ICT with desferrioxamine (Birkenfeld *et al.*, 1989).

For a comprehensive overview of the molecular mechanisms linking iron accumulation and ferroptosis to female infertility, we refer the reader to a recent review, which focuses on experimental models and pre-clinical evidence (Zhang *et al.*, 2024).

### Systemic effects

IO contributes to dysfunction of the HPG axis by affecting other endocrine glands as well as through non-endocrine mechanisms. Among the endocrine complications, diabetes mellitus, resulting from pancreatic β-cell siderosis, affects up to 35% of patients with thalassaemias (El-Samahy *et al.*, 2019; Evangelidis *et al.*, 2023) and can negatively impact HPG axis function (Thong *et al.*, 2020; Raghuraman *et al.*, 2024; Zaimi *et al.*, 2024).

IO exacerbates susceptibility to infections (Issaragrisil *et al.*, 1987; Ganz, 2018), induces a pro-inflammatory state (Kernan and Carcillo, 2017), and contributes to nutritional deficiencies (Fung, 2010), all of which may indirectly impair gonadal function in both sexes. Chronic hypoxia from long-standing anaemia has been suggested as a mechanism for gonadal failure (George *et al.*, 1997), though its relevance has diminished with optimized transfusion protocols.

Finally, organ dysfunctions secondary to IO, such as liver and heart failure, frequently contribute to functional hypogonadism (Spaziani *et al.*, 2023a). These systemic effects, summarized in Table 2, highlight the multifaceted impact of IO on gonadal health and the need for comprehensive management strategies.

**Table 2.** Summary of systemic effects of iron overload disorders.

System/organ	Primary effects	Pathophysiological mechanism	Prevalence (if available)	PMID
**Hypothalamus–pituitary gland**	Hypogonadism (hypogonadotropic or mixed forms)	Iron deposition in the HPG axis Gonadotroph toxicity	Up to 60% in men with β-TM	19604241 28732253
	Hypothyroidism	Iron accumulation in thyroid follicles	∼39% in β-TM	26957357
	GHD	Siderosis of the hypothalamus–pituitary axis	Up to 50%	11866338 29755708
**Reproductive system**	Male infertility	Peritubular iron deposition and oxidative stress affecting spermatogenesis	Azoospermia: 17.6% Other sperm abnormalities: 35.9%	22496346 20522002
	Reduced testosterone levels	Direct Leydig cell toxicity, altered steroidogenesis	Linked to hypogonadism	31233037 36558426
	Female infertility	Ovarian iron deposition, granulosa cell toxicity, reduced ovarian reserve	AMH reduction: ∼45–55%	26536400 22035015 28732253
	Endometrial dysfunction	Glandular haemosiderosis impacting implantation potential	Observed in women with serum ferritin ∼3500 ng/ml	2759323
**Skeletal system**	Osteoporosis and reduced bone mass	Hypogonadism and GHD Direct iron toxicity on bone metabolism	Up to 60%	27309522 19912219
**Cardiovascular system**	Cardiomyopathy	Myocardial siderosis and oxidative stress	15–33%	11913479 16604332
	Thrombosis	Altered coagulation pathways	Significant risk in splenectomized patients	20001616
**Liver**	Chronic liver disease	Hepatic siderosis, leading to fibrosis and cirrhosis	∼30% in β-TM	2732293
	Dysregulated glucose and lipid metabolism	Iron-induced oxidative stress and mitochondrial dysfunction	Common in hypogonadal individuals	39587544
**Pancreas**	Diabetes mellitus	Iron-induced β-cell siderosis and oxidative stress	>5%	7634497
**Immune system**	Increased susceptibility to infections	Iron promotes pathogen proliferation and reduces immune efficiency	Variable	3689939 29147843

β-TM, β-thalassaemia major; AMH, anti-müllerian hormone; GHD, growth hormone deficiency; HPG, hypothalamic–pituitary–gonadal; PMID, PubMed Identifier.

Taken together, these findings underscore the extensive impact of IO on reproductive health in both sexes, highlighting the need for rigorous monitoring of the function of the HPG axis.

## Methods

A structured literature search was conducted across PubMed, Scopus, and Web of Science databases, including studies published up to May 2025. The search strategy employed the following combination of keywords: ‘iron’ OR ‘overload’ OR ‘thalassemia’ OR ‘thalassaemia’ OR ‘transfusion*’ OR ‘hemochromatosis’ OR ‘haemochromatosis’ OR ‘hemosiderosis’ OR ‘haemosiderosis’ OR ‘ferroptosis’ AND ‘female*’ OR ‘woman’ OR ‘women’ OR ‘man’ OR ‘men’ OR ‘female*’ OR ‘male*’ AND ‘infertility’ OR ‘fertility’ OR ‘reproduction’ OR ‘reproductive’ OR ‘pregnancy’ OR ‘pregnant’ OR ‘hypogonadism’ OR ‘hypogonadal’ OR ‘damage’ OR ‘testicular’ OR ‘testis’ OR ‘testes’ OR ‘spermatogenesis’ OR ‘ovary’ OR ‘ovaries’ OR ‘ovarian’ OR ‘failure’ OR ‘insufficiency’ OR ‘menopause’ OR ‘testosterone’ OR ‘estrogens’ OR ‘oestrogens’ OR ‘pituitary’ OR ‘hypothalamic’ OR ‘hypothalamus’ OR ‘ART’ OR ‘HRT’ OR ‘ERT’ OR ‘sexual dysfunction’ OR ‘erectile dysfunction’.

This review follows a narrative format and was not registered as a systematic review. However, to enhance the transparency and consistency of the synthesis, we employed structured selection and appraisal criteria. Studies were screened and selected based on their relevance to clinical outcomes, mechanistic insights, and therapeutic implications in individuals with IO. Particular attention was given to methodological quality, diagnostic definitions, and robustness of reported reproductive endpoints. Studies of poor-quality or lacking clear reproductive outcomes were not excluded *a priori*, but this was explicitly discussed in their limitations.

In addition to the qualitative synthesis, we performed a structured extraction of numerical data from studies reporting on the weighted mean prevalence (alongside ranges, with the exclusion of case series and reports) of reproductive abnormalities, including prevalence of hypogonadism, semen alterations and menstrual disturbances (i.e. primary and secondary amenorrhoea), and the weighted age at menarche, as well as data concerning pregnancy outcomes in adult patients with IO conditions. These data were aggregated to provide a descriptive overview of the reproductive burden across sexes, supporting the interpretation of clinical patterns and pathophysiological mechanisms presented in the narrative.

Throughout the manuscript, we use the terms TDT and non-transfusion-dependent thalassaemia (N-TDT) to refer to clinical phenotypes defined by the requirement for regular blood transfusions, in accordance with contemporary classifications. Historical genotype-based terms such as TM and TI, which were widely used in earlier literature, are retained when reporting data from original studies or when explicitly referring to genetic classifications. This approach is intended to maximize terminological consistency while preserving fidelity to the source literature.

## Hypogonadism

### Prevalence and characteristics

Dysfunction of the HPG axis in IO disorders significantly impacts gonadal function, with hypogonadism being the most frequent complication of IO disorders. Across both male and female cohorts, the reported prevalence of reproductive dysfunction in IO disorders shows wide variability. This heterogeneity reflects substantial differences among published studies in terms of underlying conditions (e.g. transfusion-dependent vs non-transfusion-dependent disorders), severity and duration of iron burden, chelation protocols, age distribution, study design, and diagnostic criteria. In addition, many studies originate from tertiary referral centres, potentially enriching for more severe clinical phenotypes. Accordingly, the weighted mean prevalences presented in Tables 3, 4, and 5 should be interpreted as descriptive indicators of overall disease burden, with the implicit limitations of residual bias and limited comparability across cohorts to be considered when extrapolating these data to individual patients or contemporary clinical settings.

**Table 3.** Hypogonadism in transition age and adult men with iron overload disorders.

First author, year	Condition	N	Study design	Hypogonadism (%)	PMID
**β-TM/β-TI**					
Bajoria, R., 2011	β-TM	30	Prospective study	63.3	22035015
De Sanctis, V., 2008	β-TM, β-TI	28	Prospective study	3.6	19337176
Papadimas, J., 2002	β-TM, β-TI	67	Cross-sectional study	55.2	17018446
Papadimas, J., 1996	β-TM	30	Case–control study	40	8921062
**TDT/N-TDT**					
Chen, M. J., 2018	TDT	21	Cross-sectional study	33.3	29166371
Chern, J. P. S., 2003	TDT	11	Cross-sectional study	72.7	14608198
Singer, S. T., 2015	TDT	7	Case series	42.9	26044409
**HC/SCA**					
Fabio, G., 2007	HC	1	Case report	100	16960153
Gharwan, H., 2014	SCA	1	Case report	100	24936546
Hamer, O. W., 2001	HC	1	Case report	100	11507369
Oehninger, S., 1998	HC	1	Case report	100	9764650
Selvais, P. L., 1993	HC	1	Case report	100	8497447
Siemons, L. J., 1987	HC	1	Case report	100	8497447
Tweed, M. J., 1998	HC	1	Case report	100	9552844
Vogt, H. J., 1987	HC	1	Case report	100	3122599
**Total**		202	**Weighted mean**	47.0	

β-TI, β-thalassaemia intermedia; β-TM, β-thalassaemia major; HC, haemochromatosis; (N)-TDT, (non)-transfusion-dependent thalassaemia; PMID, PubMed Identifier; SCA, sickle cell anaemia.

**Table 4.** Azoospermia and other semen analysis alterations in transition age and adult men with iron overload disorders.

First author, year	Condition	N	Study design	Azoospermia (%)	Other SAAs (%)	Notes	PMID
**β-TM/β-TI**							
De Sanctis, V., 2008	β-TM, β-TI	28	Prospective study	14.2	57.1		19337176
De Sanctis, V., 2016	β-TM	11	Prospective study	60	40		26740862
Papadimas, J., 1996	β-TM	30	Case–control study	0	12.5		8921062
Perera, D., 2002	β-TM	6	Case–control study	0	66.6		12093845
De Sanctis, V., 2011	β-TI	16	Case series	0	25		21705984
**TDT/N-TDT**							
Chen, M. J., 2018	TDT	21	Cross-sectional study	7	46.2		29166371
De Sanctis, V., 2019c	TDT, N-TDT	966	Survey	17.9	37.7	24.8% married/lived with partner 15.5% fathered via natural conception	31580308
Singer, S. T., 2015	TDT	7	Case series	42.9	28.6		26044409
**HC/SCA**							
Gharwan, H., 2014	SCA	1	Case report	100	0		24936546
Oehninger, S., 1998	HC	1	Case report	100	0		9764650
Siemons, L. J., 1987	HC	1	Case report	100	0		3624416
**Total**		1088	**Weighted means**	17.6	37.5		

β-TI, β-thalassaemia intermedia; β-TM, β-thalassaemia major; HC, haemochromatosis; (N)-TDT, (non)-transfusion-dependent thalassaemia; PMID, PubMed Identifier; SAAs, sperm analysis alterations; SCA, sickle cell anaemia.

Studies are listed according to condition, study design, and alphabetical order. SAAs, comprising oligo-, astheno-, and teratozoospermia were defined according to the respective ‘WHO laboratory manual for the examination and processing of human semen’ fifth percentiles referenced in the original studies.

**Table 5.** Age at menarche, prevalence of primary and secondary amenorrhoea, and ferritin cut-offs in transition age and adult women with iron overload disorders.

First author, year	Condition	n	Study design	Menarche (yrs)	PA (%)	SA (%)	Notes	PMID
**β-TM/β-TI**								
Farmaki, K., 2009	β-TM	26	Clinical trial	/	35.0	59.0	Ferritin levels: 2.696 ± 660 ng/ml (PA group), 1.501 ± 273 ng/ml (SA group)	19912219
Al-Rimawi, H. S., 2005	β-TM	25	Prospective study	11.9 ± 1.5	32.2	5.5		17027358
Chatterjee, R., 1993	β-TM	15	Prospective study	14.2 ± 0.2	0.0	100.0	Age at amenorrhoea 20.6 ± 1.6	8222291
Berkovitch, M., 2000	β-TM	13	Retrospective study	/	7.7	25.0		10711663
Dhouib, N. G., 2018	β-TM	12	Retrospective study	/	42.0	0.0		29755708
Yassin, M. A., 2019	β-TI	9	Retrospective study	/	0.0	22.2		31205630
De Sanctis, V., 1995	β-TM	915	Multicentre cross-sectional study	14.2 ± 2.1	59.6	23.0		7634497
Aydinok, Y., 2002	β-TM	16	Cross-sectional study	/	12.5	8.3		11866338
Bordbar, M. 2019	β-TM	388	Cross-sectional study	/	45.4	0.0		31228105
Borgna-Pignatti, C., 1985	β-TM	118	Cross-sectional study	14.3 (median)	45.2	43.5		3965675
De Sanctis, V., 2016	β-TM	23	Cross-sectional study	/	39.0	100.0	SA occurred 6.2 ± 5.2 yrs after menarche	27366710
Karabulut, A., 2010	β-TM	25	Cross-sectional study	14.5 ± 1.0	43.4	23.1		20021296
Papadimas, J., 2002	β-TM, β-TI	68	Cross-sectional study	14.2 ± 0.5	33.9	51.2		17018446
Bronspiegel-Weintrob, N., 1990	β-TM	19	Case–control study	13.8 ± 1.3	42.1	9.1		2388669
Canale, V. C., 1974	β-TM	5	Case–control study	/	20.0	50.0		4369990
Christoforidis, A., 2007	β-TM	13	Case–control study	/	0.0	15.4		17161570
Mensi, L., 2019	β-TM	21	Case–control study	/	81.0	19.0		31404374
Uysal, A., 2017	β-TM	43	Case–control study	/	18.6	40.0		28732253
Allegra, A., 1990	β-TM	10	Case series	/	100.0	0.0		2126662
Birkenfeld, A., 1989	β-TM	4	Case series	/	25.0	50.0		2759323
Kletzky, O. A., 1979	β-TM	3	Case-series	/	100.0	0.0		376543
Reubinoff, B. E., 1994	β-TM	1	Case report	18.0	0.0	100.0		7962390
**TDT/N-TDT**								
Shalitin, S., 2005	TDT	13	Retrospective study	/	30.7	33.3	Ferritin cut-off 2.500 ng/ml (based on males and females combined)	15654898
Vogiatzi, M. G., 2009	TDT, N-TDT	180	Multicenter retrospective study	15.7 ± 3.9	/	/		19604241
Fung, E. B., 2010	TDT, N-TDT	126	Multicentre cross-sectional study	15.0 ± 3.6	/	/		20547400
Chern, J. P. S., 2003	TDT	18	Cross-sectional study	15.4 ± 1.7	55.5	62.2		14608198
**(J)HC/DBA**								
Kelly, T. M., 1984	HC	23	Retrospective study	/	0.0	0.0		6435491
Mascarenhas, M., 2018	DBA	1	Case report	/	100.0	0.0	Ferritin cut-off 2.223 ng/ml	28643569
Santiago de Sousa Azulay, R., 2020	JHC	1	Case report	/	100.0	0.0		32327622
**Total**		2134	**Weighted means**	14.4 ± 2.1	45.7	20.0		

β-TI, β-thalassaemia intermedia; β-TM, β-thalassaemia major; DBA, Diamond–Blackfan anemia; (J)HC, (juvenile) haemochromatosis; (N)-TDT, (non)-transfusion-dependent thalassaemia; PA, primary amenorrhoea; PMID, PubMed Identifier; SA, secondary amenorrhoea.

Studies are listed according to condition, study design, and alphabetical order.

The reproductive tract is especially vulnerable to iron toxicity during puberty; therefore, appropriate clinical follow-up and timely induction of puberty, when indicated, are essential to ensure optimal gonadal axis function in adulthood (Tenuta *et al.*, 2024). However, the detrimental impact on the gonadal axis may remain subclinical during early adolescence, becoming overt during young adulthood, or later in adult life. As such, the presence of hypogonadism may particularly affect sexual function, fertility, and psychological well-being of these patients (Delvecchio and Cavallo, 2010). For a detailed discussion of IO disorders concerning growth disorders and gonadal dysfunction in childhood and adolescence, we refer the reader to a recent review by our group (Tenuta *et al.*, 2024).

As stated above, iron deposition can affect hypothalamic–pituitary cells (resulting in hypogonadotropic or secondary hypogonadism) (Uysal *et al.*, 2017) or, less commonly, the ovaries and the testes (leading to hypergonadotropic or primary hypogonadism) (Perera *et al.*, 2010). In some patients, both sites may be affected, resulting in a mixed form of hypogonadism, characterized by low sexual hormone levels in the presence of inappropriately normal gonadotropins.

The prevalence of gonadal damage appears to correlate with IO, as reflected by serum ferritin levels (Hagag *et al.*, 2016). Indeed, in a multicentre North American study on thalassaemic syndromes, the odds of hypogonadism increased by 11.8% for every 1.000 µg/l increase in ferritin concentration (Vogiatzi *et al.*, 2009). Similarly, an Italian cohort study identified a ferritin threshold of 1.300 µg/l as predictive of new endocrinopathies, including hypogonadism, over a 5-year follow-up period (Poggi *et al.*, 2016).

However, the predictive value of ferritin remains debated since studies on small (Aydinok *et al.*, 2002; Mula-Abed *et al.*, 2008) and large cohorts of patients with transfusion-dependent β-TM failed to demonstrate a clear association between serum ferritin levels and hypogonadism (Borgna-Pignatti *et al.*, 2004; Bordbar *et al.*, 2019) or secondary amenorrhoea (De Sanctis *et al.*, 1995). These discrepancies suggest that additional factors, such as tissue-specific iron deposition, individual differences in iron metabolism, or genetic susceptibility, contribute to determining gonadal vulnerability.

### Male hypogonadism: endocrine function

#### Prevalence

Male hypogonadism is highly prevalent in patients with IO, particularly in β-TM, where rates range between 65 and 72% (Papadimas *et al.*, 2002; Chern *et al.*, 2003). Our review of 202 men with IO disorders from 15 studies yielded a weighted mean prevalence of hypogonadism of 47% (range 3.6–72.7%) (Table 3).

#### Clinical presentation

The manifestations of male hypogonadism in individuals with IO disorders are heterogeneous and influenced by age at onset, duration of exposure, and degree of endocrine dysfunction. In children and adolescents, delayed puberty (defined as absent testicular enlargement >3 ml by age 14) or arrested puberty (incomplete development within 4.5 years from pubertal start) represent hallmark features. In adults, the clinical picture is often subtle and nonspecific, frequently attributed to comorbidities or the underlying haematological condition.

The earliest and most specific signs include impaired sexual function, manifesting as reduced libido, erectile dysfunction (ED), decreased frequency of spontaneous erections, and infertility due to spermatogenic failure. Other clinical features include small testicular volume, decreased body hair, gynaecomastia, and reduced shaving frequency, especially in those with profound testosterone deficiency (Bhasin *et al.*, 2018; Corona *et al.*, 2020b; Isidori *et al.* 2022).

Androgen deficiency also exerts systemic effects on multiple target tissues. Loss of anabolic testosterone action on skeletal muscle contributes to decreased muscle mass and strength (sarcopenia), impaired physical performance, and increased risk of falls, particularly in IO, where chronic anaemia, liver dysfunction, and systemic inflammation further contribute to muscle catabolism (Rizk *et al.*, 2023). Bone health is also critically affected as testosterone promotes bone accrual both directly, through androgen receptors, and indirectly via aromatization to estradiol. In the hypogonadal male, impaired bone formation and accelerated bone resorption lead to reduced bone mineral density (BMD), cortical thinning, and increased fracture risk. The effect is more pronounced at trabecular sites, such as the vertebrae, but also affects cortical compartments (Tenuta *et al.*, 2025). A reciprocal bone–testis axis has also been described, whereby osteocalcin released by osteoblasts may influence Leydig cell function and vice-versa (Oury *et al.*, 2011; Carlomagno *et al.*, 2024).

Other systemic manifestations include fatigue, mood disturbances, and cognitive complaints, which, although nonspecific, further contribute to reduced quality of life (Isidori *et al.*, 2014; Sansone *et al.*, 2014; Bhasin *et al.*, 2018). Finally, testosterone deficiency is also associated with adverse effects on lipid metabolism and cardiovascular health, reinforcing the importance of early detection and management in this vulnerable population (Isidori *et al.*, 2005; Dean *et al.*, 2015; Rastrelli *et al.*, 2018; Corona *et al.*, 2020b).

#### Diagnosis

**Figure 1.**
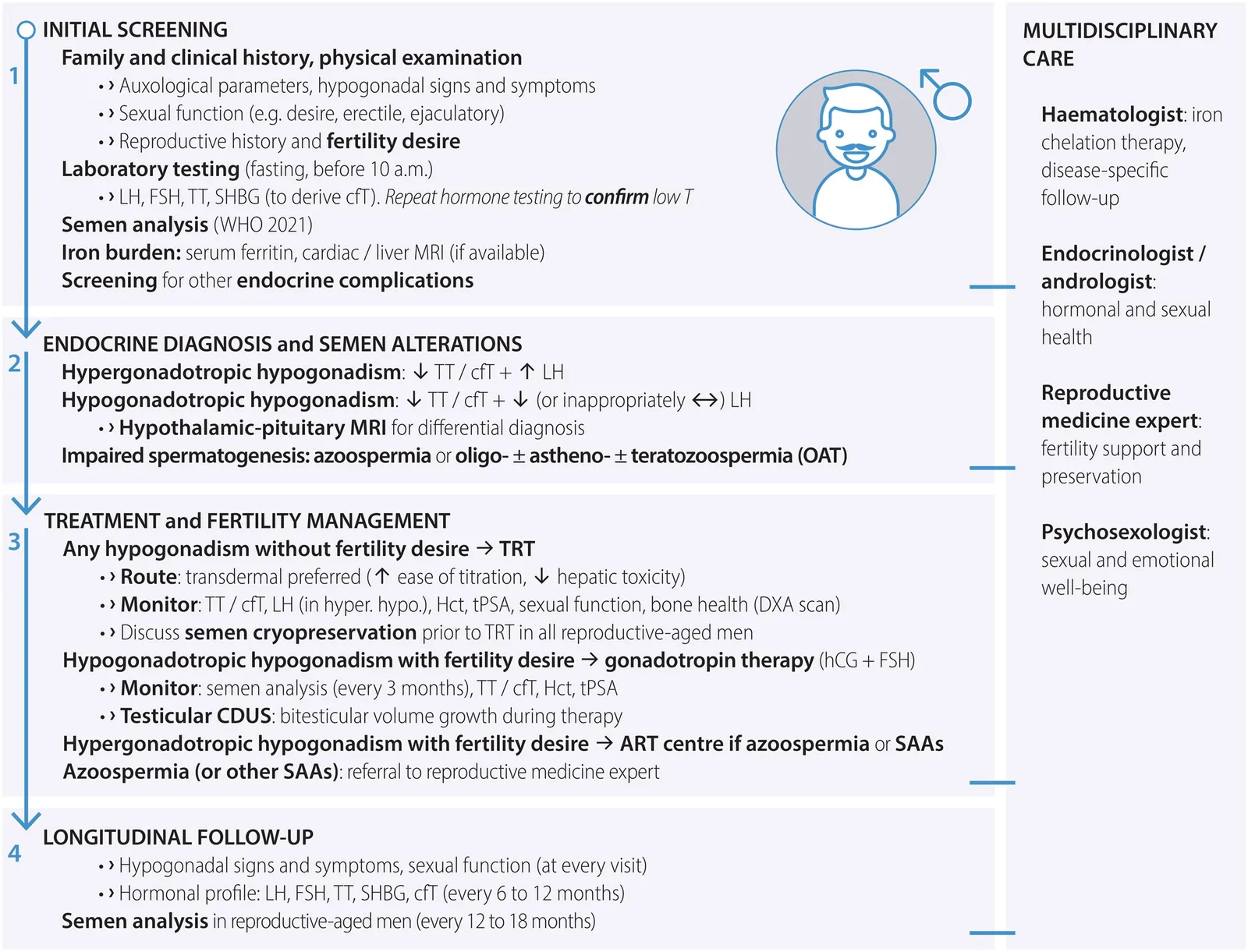
**Algorithm for clinical management and fertility preservation in men with iron overload disorders.** CDUS, colorDoppler ultrasound; cfT, calculated free testosterone; DXA, dual X-ray absorptiometry; Hct, haematocrit; MRI, magnetic resonance imaging; SAA, semen analysis abnormalities; SHBG, sex hormone-binding globulin; tPSA, total PSA; TRT, testosterone replacement therapy; TT, total testosterone.

The proposed diagnostic approach is summarized in Fig. 1. The initial evaluation should comprise a detailed clinical history (including haematological diagnosis, transfusion burden, serum ferritin levels, and ICT regimen) and an andrological physical examination to identify risk factors for male infertility or clinical signs and symptoms of hypogonadism.

Serum testosterone levels should be proactively monitored in patients with IO disorders, such as TDT, even before typical symptoms of hypogonadism become manifest (Jensen *et al.* 1997a; De Sanctis *et al.* 2004; Sansone *et al.* 2017). It is important to note that the diagnostic threshold for low serum total testosterone (TT) is not universally defined, with different scientific societies recommending different cut-offs, reflecting both population-based data and treatment efficacy thresholds (Bhasin *et al.*, 2018; Corona *et al.*, 2020a; Giagulli *et al.*, 2020; Isidori *et al.*, 2022). The European Academy of Andrology (EAA) suggests a TT level <12 nmol/l (350 ng/dl) as the upper threshold for biochemical hypogonadism, recommending TRT in symptomatic individuals below this level and strongly supporting treatment when levels fall <8 nmol/l (230 ng/dl), which are consistently associated with androgen deficiency symptoms and poorer clinical outcomes (Corona *et al.*, 2020a,b). Crucially, the EAA places particular emphasis on the calculated free testosterone (cfT) as an essential diagnostic parameter, especially in men with TT in the ‘grey zone’ between 8 and 12 nmol/l or in those with altered sex hormone-binding globulin (SHBG) levels. In such contexts, a cfT below 220 pmol/l (64 pg/ml) supports the diagnosis and guides therapeutic decisions (Corona *et al.*, 2020a,b). The Endocrine Society proposes a lower TT threshold of <10.4 nmol/l (300 ng/dl) to define hypogonadism in the presence of compatible signs and symptoms (Bhasin *et al.*, 2018). In the above-mentioned ‘grey zone’, treatment decisions must be individualized based on cfT, symptoms, comorbidities, and reproductive goals. The measurement of SHBG is even more important in IO disorders than in other conditions, since its levels are frequently elevated as a consequence of liver damage. Therefore, cfT levels may be low even when TT levels appear normal (Cundy *et al.*, 1989; Gautier *et al.*, 2011).

In addition, as a general rule, the confirmation of low testosterone levels through fasting and early morning (before 10 AM) blood tests is required for the diagnosis of hypogonadism (Isidori *et al.*, 2022). Beyond TT and cfT, hormonal profiling should include serum FSH and LH (to differentiate hypo- from hypergonadotropic hypogonadism).

Despite its prognostic value (discussed above), there are no guidelines currently supporting the routine use of pituitary MRI to evaluate the level of iron infiltration of the pituitary gland. Nonetheless, MRI of the hypothalamic–pituitary area should be considered for the differential diagnosis of hypogonadotropic hypogonadism.

Bone health assessment is essential, with dual-energy X-ray absorptiometry (DXA) recommended due to the high risk of reduced BMD and osteoporosis associated with hypogonadism, GHD, and kidney failure in this population (Farmaki *et al.*, 2010; Wong *et al.*, 2016; Ó Breasail *et al.*, 2020). Biochemical markers of bone turnover should also be evaluated, as iron-mediated oxidative stress and androgen deficiency synergistically impair bone remodelling, increasing fracture risk, even in the absence of low BMD. According to clinical context, the diagnostic workup should also include vertebral morphometric assessment to identify subclinical fractures (Burden *et al.*, 2024; Costanzo *et al.*, 2024).

#### Treatment

TRT represents the cornerstone of treatment for male hypogonadism in patients with IO disorders who are not seeking paternity, aimed at restoring physiological testosterone levels to alleviate symptoms and improve overall health. Selection of the appropriate formulation depends on patient characteristics, preferences, comorbidities, and the balance between efficacy, safety, and ease of monitoring. Several TRT options are available, including transdermal gels, long-acting intramuscular injections, and oral formulations. In patients with IO disorders, transdermal testosterone should be the preferred route of administration, as it allows for more precise dose titration (Corona *et al.*, 2020a,b) and avoids hepatic first-pass metabolism, thereby reducing the risk of liver toxicity: an important consideration in individuals with pre-existing hepatic vulnerability (Goldstein *et al.*, 2025). Moreover, transdermal formulations are ideal for younger men, as they allow early morning administration to mimic the physiological circadian rhythm of testosterone secretion (Liu, 2019; Ning *et al.*, 2025). Conversely, oral testosterone undecanoate is rarely used due to its variable and often unpredictable bioavailability (Bhasin *et al.*, 2018; Corona *et al.*, 2023). Although novel oral, injectable, and transdermal formulations with improved pharmacokinetic profiles show promise, they are not yet widely available or adopted (Miner *et al.*, 2025). TRT should be titrated according to serum testosterone levels and clinical response, aiming to restore levels within the mid-normal physiological range. Baseline evaluation should include haematocrit, liver enzymes, total PSA, lipid profile, and prostate digital exploration (if the patient is >40 years of age). Once TRT is started, and target testosterone levels are achieved, long-term follow-up should include at least annual reassessments of gonadal status, haematocrit, total PSA, and a digital rectal exam (in men >40 years), as well as comorbidities, and testosterone dosage should be adjusted accordingly. Side effects from TRT were specifically evaluated in an Italian cohort of 95 young patients with β-TM aged 17–42 years. Although gynaecomastia was reported in 43% of cases (mostly mild-to-moderate and not requiring additional treatment), this was likely related to increased aromatization of testosterone into E2, enhanced by concomitant hepatic IO and chronic liver disease, often HCV-related. Additionally, local skin or injection-site reactions occurred in ∼40% of patients (De Sanctis *et al.*, 2019b). It should be noted that, unlike the general hypogonadal population, clinically relevant TRT-induced erythrocytosis is rarely observed in men with IO disorders, who typically present with baseline anaemia and impaired erythropoiesis, often requiring regular transfusions. Standard haematological monitoring during TRT remains appropriate, but the risk of polycythaemia in this specific setting is negligible.

Testosterone also influences iron metabolism in men by inhibiting hepcidin synthesis, thereby increasing iron absorption and supporting erythropoiesis (Bachman *et al.*, 2010; Latour *et al.*, 2014; Magnussen *et al.*, 2023). While both preclinical and clinical studies in healthy individuals have shown that testosterone influences iron metabolism, primarily by reducing hepcidin levels and promoting erythropoiesis (Latour *et al.*, 2014; Dhindsa *et al.*, 2016; Hennigar *et al.*, 2020), there is no clinical evidence to suggest that hypogonadism provides a compensatory benefit in patients with IO. On the contrary, early TRT may offer additional haematological benefits, especially in transfusion-dependent patients, by stimulating erythropoiesis (Bachman *et al.*, 2010). Therefore, optimal TRT management involves personalized therapeutic strategies, ideally through a gradual increase in dosage via transdermal formulations, to balance symptomatic relief with risk mitigation (De Sanctis *et al.*, 2017b).

Beyond testosterone deficiency per se, clinicians should also address the long-term complications of hypogonadism, such as osteoporosis. Supplementation with calcium and vitamin D is recommended in deficient individuals, and antiresorptive or anabolic agents should be considered in cases of established osteoporosis, in accordance with EAA guidelines on male osteoporosis (Rochira *et al.*, 2018). Furthermore, metabolic derangements such as Type 2 diabetes mellitus and dyslipidaemia, which are common in this population, must be actively managed according to general guidelines. Statins may be used to control LDL cholesterol and reduce cardiovascular risk, while lifestyle counselling remains an essential component of care.

### Female hypogonadism: endocrine function

#### Prevalence

Hypogonadism and premature ovarian insufficiency (POI), defined as loss of ovarian function before the age of 40 years, frequently occur in females with IO disorders (Aydinok *et al.*, 2002; Tenuta *et al.*, 2024). A longitudinal cohort study spanning 10 years evaluated gonadotropin secretory dynamics in 15 menstruating females with thalassaemia, conducting 12-h gonadotropin profiling and GnRH stimulation testing at baseline and at ∼1 and 5–6 years after the onset of secondary amenorrhoea. Initially, minor differences were seen compared to healthy controls, but after 1 year of amenorrhoea, patients showed significant reductions in mean levels, amplitude, and variability of LH and FSH peaks, and increased sleep-entrained pulses. After 5–6 years, two-thirds became apulsatile, while the remainder showed marked pulse deterioration. GnRH stimulation confirmed significantly lower peak LH and FSH responses (including area under the curve) in patients compared to controls (Chatterjee *et al.*, 1993).

In an Iranian cohort (n = 388), ∼56% of women with β-TM had hypogonadism (Bordbar *et al.*, 2019): a rate confirmed by an Italian multicentre survey (n = 590, prevalence 53.3%) (De Sanctis *et al.*, 2017a), and a North American study (n = 185) showing progressive increase with ages, with hypogonadism affecting ∼52% of patients over 20 years, and the rate increasing by ∼14% for every 5 additional years (Vogiatzi *et al.*, 2009).

Our review, encompassing 2134 individuals with IO disorders from 29 studies, evidenced the presence of primary amenorrhoea in 45.7% of adolescent and adult females (range 0–81%), whereas secondary amenorrhoea occurred in an additional 20.0% of subjects (range 0–100%) (Table 5). The weighted average age at menarche was 14.4 ± 2.1 years (Table 5), which is above the mean ages reported in recent population studies: 13.4 years in Denmark (Juul *et al.*, 2006), 13.2 years in Norway (Gottschalk *et al.*, 2020), 13.1 years in the Netherlands (Talma *et al.*, 2013), 12.5 years in the USA (Anderson *et al.*, 2003), 12.4 years in Italy (Rigon *et al.*, 2010), and 12.0 years in Portugal (Queiroga *et al.*, 2020).

The wide variability in reported prevalences reflects a combination of methodological and clinical factors. These include differences in underlying diagnoses and severity of iron accumulation, variability in chelation regimens (with more effective agents becoming available only in more recent years), selection and reporting biases, and heterogeneity in study design and clinical settings (e.g. retrospective vs prospective cohorts, paediatric vs adult populations). The majority of available studies in adult women focus on β-TM, the most severe form of IO disorders, whereas only a few have investigated rarer conditions. In a relatively large study population (n = 354), the highest prevalence was observed in patients with β-TM (51.3%), reflecting the greater transfusion burden, whereas lower rates were reported in β-TI (9.3%) and E-β thalassaemia (14.3%), with no cases identified in haemoglobin H or Constant Spring diseases (Vogiatzi *et al.*, 2009). Similarly, a study of 49 women with β-TM confirmed that hypogonadotropic hypogonadism was more prevalent in those with more severe β- and α-globin chain defects (Jensen *et al.*, 1997a). An international cross-sectional study on 522 patients (262 females) further supported the low prevalence of hypogonadism in β-TI (9.8%) (Karimi *et al.*, 2020). Consistently, the lowest prevalence was observed in N-TDT, with only 2% of cases affected (Marsella *et al.*, 2018).

Female hypogonadism in HH has been less studied. A large study (n = 1140) compared *HFE-* to non-*HFE-*related HH and revealed that non-*HFE* cases had an earlier onset and a more severe clinical course. Hypogonadism was more common in non-*HFE* forms, particularly juvenile HH (related to mutations in *HJV* and *HAMP*, 70%), compared to carriers of *TFR2* (26%) and *HFE* mutations (14% in probands, 7% in non-probands), even after adjusting for age, sex, and serum ferritin. Since the above-mentioned genes, with the exception of *HFE*, are not expressed in the ovary, these findings suggest that the observed differences in the frequency of hypogonadism are likely to be associated with the timing and extent of iron accumulation. Indeed, an earlier and more rapid iron accumulation in juvenile forms has a stronger impact on the endocrine system, leading to a higher prevalence of hypogonadism (Sandhu *et al.*, 2018).

#### Clinical presentation

Similar to males, also in females, hypogonadism may differ in its presentation as a result of the age and degree of gonadal dysfunction. Specifically, during childhood or adolescence, it may present as either delayed (absence of thelarche by age 13) or arrested puberty (incomplete pubertal development within 4.5 years from pubertal start), and women with fully developed secondary sexual characteristics may experience primary amenorrhoea (absence of menarche within 5 years from pubertal start, or by 16 years of age), whereas previously menstruating women might incur subtle menstrual irregularities progressing to overt secondary amenorrhoea. These manifestations are often accompanied by a range of nonspecific symptoms, including fatigue, reduced libido, and vasomotor complaints such as hot flushes or night sweats, which may be mistakenly attributed to other causes or under-recognized. Beyond menstrual irregularities, estrogen deficiency may lead to progressive declines in BMD and mood instability, cognitive changes, and genitourinary symptoms, including vaginal dryness or dyspareunia. These features, while often subtle, contribute to impaired quality of life and are frequently overlooked unless systematically investigated.

#### Diagnosis

**Figure 2.**
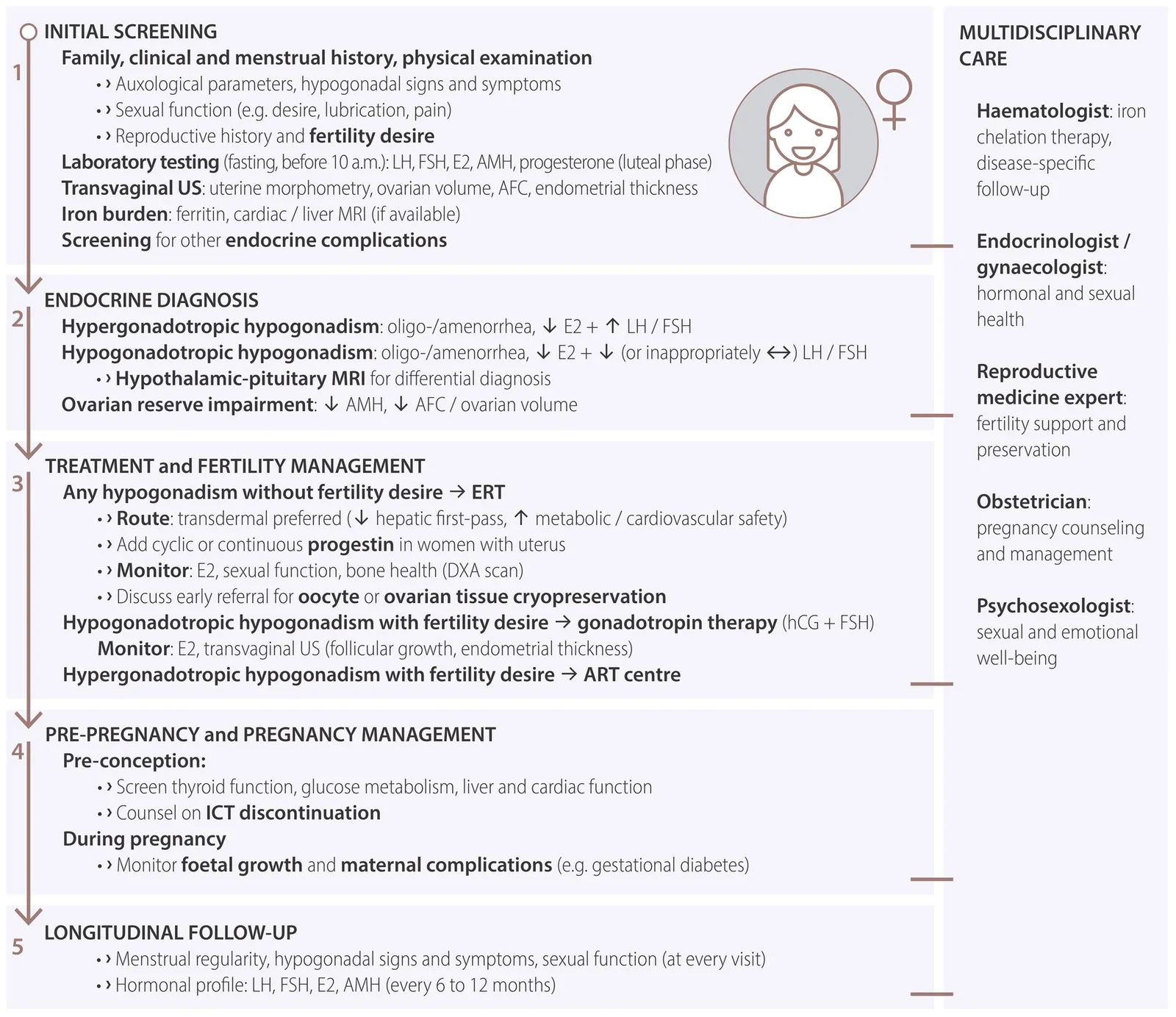
**Algorithm for clinical management and fertility preservation in women with iron overload disorders.** AFC, antral follicle count; AMH, anti-Müllerian hormone; DXA, dual X-ray absorptiometry; E2, estradiol; ERT, estrogen replacement therapy; ICT, intensive chelation therapy; MRI, magnetic resonance imaging; TT, total testosterone; US, ultrasound.

The diagnostic approach to pubertal disorders and amenorrhoea in this clinical setting mirrors that applied to the general population and is summarized in Fig. 2. As in males, the initial assessment involves a thorough clinical history, with particular focus on pubertal development milestones, age at menarche, menstrual cycle regularity, and symptoms suggestive of estrogen deficiency. A menstrual calendar can be a valuable tool to document cycle patterns and identify abnormalities over time. Physical examination should include a full general and gynaecological assessment, noting the development of secondary sexual characteristics, body composition, and any signs indicative of hypestrogenism. In peri-pubertal and young post-pubertal patients, pelvic examination and ultrasonography can provide valuable information regarding uterine development, ovarian volume and morphology, and endometrial thickness (Singer *et al.*, 2010, 2011).

Endocrine assessments should ideally be performed during the early follicular phase (i.e. at Days 2–5 of the menstrual cycle, or after an interval >40 days without menstruation), or after suspending ERT for at least 2–3 months in amenorrhoeic individuals. An early-morning fasting hormone panel should include serum FSH, LH, E2, prolactin, and AMH concentrations (to assess ovarian reserve, discussed below in the section on ‘Reproductive function’), which together help differentiate between hypogonadotropic and hypergonadotropic hypogonadism.

As in males, hypothalamic–pituitary MRI is part of the exams used for the differential diagnosis of central hypogonadism, whereas routine MRI with targeted imaging for iron deposition is not recommended by the current guidelines.

BMD assessment via DXA is strongly recommended in hypogonadal women of any age. Specifically, patients experiencing hypogonadism during childhood and adolescence, or before the end of transition age (defined as the phase between the end of puberty and young adulthood, usually considered to last until ∼25 years of age (Grugni *et al.*, 2021; Feola *et al.*, 2023)) will miss peak bone mass achievement; on the other side, women with later onset hypogonadism will be exposed to an earlier decline of BMD. Both cases will result in a markedly increased risk of osteopenia and/or osteoporosis (Chemaitilly *et al.*, 2017; Dhakate *et al.*, 2023).

#### Treatment

Timely recognition and treatment of hypogonadism in women with IO is essential to alleviate symptoms related to E2 deficiency, prevent long-term complications, and improve quality of life. ERT remains the cornerstone of treatment in adult women with hypogonadism.

ERT regimens should be individualized, considering patient age, severity of IO, extent of HPG axis disruption, liver function, pregnancy plans, and personal preferences. In women with an intact uterus, estrogen is administered in combination with a progestin to protect the endometrium (Panay *et al.*, 2024). The preferred estrogen is 17β-estradiol, due to its safer profile regarding lipid metabolism and coagulation compared to ethynilestradiol and its facilitation of precise hormone level monitoring. It also benefits peak bone mass acquisition in the transition age (Crofton *et al.*, 2010). Among progestins, micronized progesterone is favoured for its superior cardiovascular safety profile (Mueck, 2012) and overall tolerability (Davey, 2013).

Common regimens include continuous combined or cyclical combined therapy, where a progestin is co-administered for 12–14 days each month to ensure withdrawal bleeding (Lambrinoudaki *et al.*, 2021), with the latter offering a potentially lower risk of endometrial hyperplasia and malignancy (Furness *et al.*, 2012).

In patients with IO, transdermal estrogen administration should be strongly preferred, as it bypasses first-pass hepatic metabolism, thereby reducing the impact on liver function, which is particularly important in thalassaemic patients predisposed to hepatic complications. This route also ensures more stable serum estradiol concentrations, exerts neutral effects on circulating bioavailable IGF-1 (particularly relevant in young adults, e.g. for peak bone mass achievement), and is associated with a more favourable profile in terms of blood pressure, lipid metabolism, and inflammatory markers (Divasta and Gordon, 2010). For these reasons, oral contraceptives are not recommended for ERT in these patients, as they may increase biliary lithogenicity, which is of concern in thalassaemic patients prone to liver dysfunction and haemolysis, with the prevalence of gallbladder disease estimated at ∼30% (De Sanctis *et al.*, 2017b). Progestins can be administered orally, transdermally, or via intrauterine devices, with no route showing clear superiority (Panay *et al.*, 2024).

Although hormone therapy has been historically associated with increased risk of thromboembolism, stroke, cardiovascular events, and hormone-sensitive cancers such as breast and endometrial carcinoma (De Sanctis *et al.*, 2017a), these risks mostly derive from studies in older women undergoing menopausal hormone therapy, rather than ERT for hypogonadotropic hypogonadism or POI (Weiderpass *et al.*, 1999; Ewertz *et al.*, 2005; Canonico *et al.*, 2014; Zhu *et al.*, 2020; Panay *et al.*, 2024). In young hypogonadal women, the use of physiological estradiol doses, aimed at achieving serum concentrations within the mid-follicular range (∼120 pg/ml), is generally considered safe and is not associated with an increased risk of breast cancer before the age of 50 years (Wu *et al.*, 2014; Panay *et al.*, 2024). These considerations extend to women who have undergone splenectomy, in whom any increased thrombotic risk appears to be primarily driven by the underlying haematological condition, including chronic haemolysis, thrombocytosis, and systemic inflammatory activation, rather than by ERT per se (Swinson *et al.*, 2022).

Treatment should be personalized in every patient, regularly monitored, and adjusted over time to balance overall patient well-being, symptom control, and metabolic and bone health.

## Sexual function

### Male sexual function

Sexual dysfunction, particularly ED, is common in men with IO disorders and results from a complex interplay of factors. Among these, hypogonadism plays a central role, as testosterone is essential for maintaining libido, erectile capacity, and overall sexual function (Papadimas *et al.*, 2002; Jannini *et al.*, 2010; Bordbar *et al.*, 2019). By contrast, alterations in spermatogenesis primarily reflect testicular damage and oxidative stress and may be clinically silent from a sexual standpoint.

High rates of sexual symptoms, including reduced libido, erectile and ejaculatory dysfunction, have been consistently reported in TDT, where chronic transfusional IO leads to pituitary siderosis, acquired hypogonadotropic hypogonadism, and, in a subset of patients, primary testicular damage (De Sanctis *et al.*, 2016). Recent reviews estimate that ED may affect ∼50–70% of men with TDT, reflecting the cumulative burden of iron toxicity, oxidative stress, and long-standing endocrine dysfunction (Roidos *et al.*, 2024). Longitudinal data are more robust in N-TDT, in which a large population-based study demonstrated a markedly increased risk of ED compared with healthy controls, with an adjusted hazard ratio of 4.56 (Chen *et al.*, 2015). Although the pathogenic background differs, being driven by chronic anaemia, ineffective erythropoiesis, and progressive iron loading rather than transfusional exposure, these findings confirm that ED represents a clinically relevant complication across the thalassaemia spectrum, rather than being restricted to a single disease phenotype.

Beyond androgen deficiency, additional abnormalities may further exacerbate ED in this population. Thyroid dysfunctions, particularly central hypothyroidism, which may occur in IO, can impair vascular tone and endothelial function, thereby contributing to ED independently of testosterone status (De Sanctis and Giovannini, 2011; Sansone *et al.*, 2014, 2015; Seow *et al.*, 2021; Yu, 2021).

A major underlying mechanism involves oxidative stress caused by iron-induced ROS. These ROS impair erection by depleting nitric oxide and inactivating soluble guanylate cyclase, a key mediator in nitric oxide (NO)-dependent vasodilatation, thereby directly impairing erectile function (Burnett *et al.*, 1992; Decaluwé *et al.*, 2017). Moreover, IO may also cause peripheral nerve damage, which further contributes to sexual dysfunction. In advanced stages of HH, axonal degeneration and autonomic neuropathy have been documented (Seravalle *et al.*, 2016), compromising both the sensory feedback and autonomic control (Wouthuis *et al.*, 2010; Rance and Chisari, 2016) necessary for erectile and ejaculatory functions. Although evidence remains limited, disruptions in the neural reflex arcs responsible for penile and pelvic innervation may also play a role in ejaculatory disorders (Pique *et al.*, 2021).

The above-listed mechanisms act synergistically, explaining the high prevalence of ED and other sexual dysfunctions in men with IO disorders. In clinical practice, however, sexual symptoms do not mirror the severity of spermatogenic impairment: patients with severe oligozoospermia or azoospermia may report preserved libido and erectile function, whereas others with marked sexual dysfunction may retain residual fertility potential. Therefore, comprehensive management should incorporate a thorough assessment of vascular and neurological functions to identify underlying damage. A tailored, multidisciplinary approach, potentially incorporating psychosexological support, TRT when necessary and appropriate, and the use of phosphodiesterase type 5 inhibitors, can help optimize sexual outcomes and enhance overall quality of life.

The psychological burden associated with chronic disease and frequent medical interventions further exacerbate sexual dysfunction in this population. Heightened stress, anxiety, and emotional distress can amplify sexual dysfunction, and without timely intervention, mild or subclinical symptoms may progress to more severe and persistent conditions (Jannini *et al.*, 2006; Colonnello *et al.*, 2021). Psycho-sexological support is thus a crucial but often underutilized component of care. Addressing the psychological and emotional dimensions of sexual dysfunction alongside physical contributors requires an integrated, holistic strategy.

### Female sexual function

Female sexual dysfunction has not been specifically investigated in the context of IO disorders; however, clinically relevant insights can be drawn from analogous conditions characterized by hypogonadism and ovarian dysfunction. In hypogonadal women, reduced estrogen levels commonly lead to vaginal dryness, which may result in dyspareunia (pain during intercourse), as well as diminished libido and impaired sexual arousal. These issues particularly impact young women, in whom sexual dysfunction can disrupt psychosocial development, self-esteem, and intimate relationships. The psychological and relational burden may be especially pronounced in individuals with arrested puberty or primary amenorrhoea (Varughese *et al.*, 2021). In hypogonadal women, ERT, aimed at restoring estrogen levels to an age-appropriate range, is effective in improving all domains of sexual function in women with POI (Panay *et al.*, 2024). The use of vaginal estrogens may be considered as add-on therapy for women with persistent genitourinary symptoms, such as vulvovaginal atrophy (Panay *et al.*, 2024). Furthermore, women with persistent low libido, suggestive of hypoactive sexual desire disorder, may benefit from systemic testosterone therapy (Lambrinoudaki *et al.*, 2021; Panay *et al.*, 2024) and/or from psychosexual therapy (Fruhauf *et al.*, 2013).

Beyond these clinical considerations, it should be acknowledged that female sexual health has not been systematically evaluated in women with IO disorders. To date, no studies have assessed domains such as desire, arousal, lubrication, orgasm, satisfaction, or pain using validated psychometric instruments, including the Female Sexual Function Index, in this population.

It should also be stressed that sexual dysfunction in this context extends beyond physical symptoms, significantly affecting emotional well-being and interpersonal dynamics. Acknowledging and addressing these aspects is essential for comprehensive care. Although more research is needed, clinical experience suggests that appropriate ERT and psychosexual counselling can meaningfully improve sexual function and quality of life in affected women.

Taken together, the absence of targeted clinical data represents a major knowledge gap. Future studies in women with IO should integrate validated sexual function instruments with hormonal profiling, assessment of ovarian reserve, and quantification of systemic and tissue iron burden to clarify whether iron excess affects not only reproductive function (e.g. hypogonadism or POI), but also broader aspects of female sexual wellbeing.

## Reproductive function

Disruption of the HPG axis in IO disorders not only leads to hypogonadism and sexual dysfunction but also significantly impairs reproductive potential. Effective strategies to safeguard fertility include preventive measures, systematic pre-conceptional evaluation of both partners, and targeted therapeutic interventions to address infertility. A structured multidisciplinary approach is essential in this regard and should involve endocrinologists, haematologists, andrologists, gynaecologists, radiologists, and mental health specialists (Cassinerio *et al.*, 2017).

In our review of 1088 men with IO disorders from 11 studies, the weighted mean prevalence of azoospermia was 17.6% (range 0–60.0%), with additional sperm abnormalities observed in 37.5% of cases (range 12.5–66.6%) (Table 4). In women, as discussed above, the weighted overall prevalence of (primary and secondary) amenorrhoea totalled over 65% (Table 5). However, while studies reporting ART outcomes in individuals with IO disorders are available (Table 6), no data exist on the overall prevalence of infertility or the proportion of affected individuals actively seeking care at ART centres.

**Table 6.** Pregnancy outcomes data in women with iron overload disorders.

First author, year	Conditions	Pregnancies	Women	ART	Miscarriage	C-section	Prematurity	Time of delivery	Birth weight	PMID
**β-TI/β-TM/HbE-β/HbH**										
Bajoria, R., 2009	β-TM	14	11	100%	0%	73.0%	–	–	2.50	20001616
Cassinerio, E., 2017	β-TM	44	30	54.1%	0%	89.2%	8.1%	37.0 ± 2.0	2.59 ± 0.64	28321530
Daskalakis, G. J., 1998	β-TM	9	9	11.1%	11.1%	100%	0%	37.7 ± 0.5	2.84 ± 0.32	9763058
Fozza, C., 2017	β-TM	20	15	60.0%	25.0%	93.3%	75.0%	29–36	–	28992618
Gulino, F. A., 2013	β-TM	6	5	0%	0%	100%	0%	40	2.85	24020709
Jensen, C. E., 1995	β-TM	16	11	25.0%	0%	76.9%	23,0%	37.6 ± 3.4	2.82 ± 0.83	7654640
Mancuso, A., 2008	β-TM	5	5	60,0%	0%	80.0%	20.0%	37.2 ± 1.3	2.64 ± 0.31	18182786
Tampakoudis, P., 1997	β-TM	5	5	0%	0%	80.0%	20,0%	38.4 ± 1.3	2.54 ± 0.39	9306104
Tuck, S. M., 2005	β-TM	29	22	41.4%	6.9%	75.0%	16.7%	–	3.24	16339678
Aessopos, A., 1999	β-TM/β-TI	22	19	0%	4.5%	100%	–	37–40	3.08 (2.6–3.65)	9988801
Al-Riyami, N., 2014	β-TM/β-TI	15	10	7,0%	7%	57.0%	0%	38.6 ± 0.9	2.60 ± 0.20	25097768
Ansari, S., 2006	β-TM/β-TI	62	32	0%	19.4%	24.0%	10.0%	–	2.68 (1.5–3.85)	16326410
Origa, R., 2010	β-TM/β-TI	75	57	12,0%	5.3%	78.4%	28.4%	–	–	19903676
Toumba, M., 2008	β-TM/β-TI	161	100	–	4.3%	–	13.3%	–	2.70	18720627
Virot, E., 2022	β-TM/β-TI	75	37	14.3%	21.3%	53.6%	21.4%	37.7	2.78	34668980
Vlachodimitropoulou, E., 2018	β-TM/β-TI	14	14	64.3%	0%	57.1%	–	33–40	–	29390937
Nassar, A. H., 2008	β-TI	83	44	–	22.9%	72.7%	31.8%	36.5 ± 3.1	2.55 ± 0.62	29390937
Roumi, J. E., 2017	β-TI	85	48	2.4%	7.1%	59.5%	2.5%	37 ± 2.4	2.82 ± 0.70	28247418
Voskaridou, E., 2014	β-TI	60	34	8.3%	8.3%	67.3%	0%	37.0 ± 3.0	2.70 ± 0.40	24889414
Luewan, S., 2009	HbE-β	54	54	–	0%	27.8%	35.2%	36.4 ± 3.0	–	19084837
Su, J.-Y., 2023	HbH	28	28	–	0%	35.7%	–	–	2.95 ± 0.47	36808349
Thompson, A. A., 2013	Mixed	129	72	24.4%	12.4%	–	11,0%	–	–	23757266
**DBA/HC/HDA**										
Alter, B. P., 1999	DBA	25	29	–	3.4%	25.0%	–	38.0 (33–40)	2.62 (1.42–3.51)	10520024
Faivre, L., 2006	DBA	64	26	–	45.2%	–	11.3%	36.3 ± 5.6	2.69 ± 1.3	16537118
Niu, C., 2023g	HC	2408	2408	–	–	19.7%	6.9%	–	–	37774920
Shalev, H., 2008	HDA	28	6	0%	7.1%	35.7%	23.1%	38.4 ± 3.6	–	18573172
**Total**		3536	3131	19.4%	11.2%	29.7%	9.8%	37.1 ± 3.1	2.64 ± 0.68	

We report means ± standard deviations, except for when only medians or ranges are available. Time of delivery is reported in weeks, and birth weight in kilograms. The prevalence of ART includes patients undergoing controlled ovarian stimulation, IUI, FIVET, and/or ICSI. Case reports are excluded. Weighted means are reported for pregnancy outcomes at the bottom of the table. Studies are listed according to condition and alphabetical order.

β-TI, β-thalassaemia intermedia; β-TM, β-thalassaemia major; DBA, Diamond-Blackfan anaemia; HbE-β, haemoglobin E disease-β-thalassaaemia; HbH, haemoglobin H disease; HC, haemochromatosis; HDA, hereditary dyserythropoietic anaemia; PMID, PubMed Identifier.

### Preventive measures for future fertility

Despite improved survival and reduced systemic complications, fertility remains especially vulnerable in patients with IO disorders due to the early onset and progressive nature of gonadal and pituitary damage, highlighting the need for proactive and timely fertility preservation strategies (Castaldi and Cobellis, 2016; Tenuta *et al.*, 2024).

In male patients, preserving fertility requires a tailored approach based on the patient’s age and stage of development. Post-pubertal patients should be offered sperm cryopreservation as early as feasible, before azoospermia develops, considering the frequent occurrence of hypogonadotropic hypogonadism (De Sanctis *et al.*, 2017b) and testicular oxidative damage beginning in adolescence (Chen *et al.*, 2018). Ejaculation-based semen collection remains the standard, and for those who have spermatozoa in the ejaculate, sperm cryopreservation represents the gold standard (Ewy *et al.*, 2025). In addition, in young azoospermic subjects, testicular sperm extraction should be attempted to retrieve and cryopreserve spermatozoa (Ewy *et al.*, 2025). In peri-pubertal males unable to provide semen samples, penile vibratory stimulation and electroejaculation may be considered as last-resort options. However, these techniques raise potential ethical and psychological concerns due to their invasive nature and should be considered only in subjects with favourable testicular volumes (i.e. above 6–7 ml) (Hagenäs *et al.*, 2010) and in the absence of additional risk factors for azoospermia or oligoasthenoteratozoospermia (OAT) (Yu, 2019; Duffin *et al.*, 2024). Testicular tissue cryopreservation also represents a promising experimental option in prepubertal patients. The recently published ESHRE good practice recommendations for fertility preservation in pre- and peri-pubertal males provide detailed recommendations on this technique, which consists of freezing small fragments of immature testicular tissue containing spermatogonial stem cells, with the aim of restoring fertility in adulthood through future technologies such as *in vitro* spermatogenesis or autologous tissue transplantation (Mitchell *et al.*, 2025).

In post-pubertal female patients, oocyte cryopreservation following ovarian stimulation can ensure fertility preservation prior to significant decline of the ovarian function (Singer *et al.*, 2011; Mensi *et al.*, 2019). Given the hypogonadotropic (or mixed) hypogonadism often observed in these patients, ovarian stimulation protocols with gonadotropins should be personalized in function of the specific endocrine status and ovarian reserve.

In prepubertal females undergoing haematopoietic stem cell transplantation, ovarian tissue cryopreservation is increasingly offered (Shapira *et al.*, 2014; Matthews *et al.*, 2018). However, in patients affected by IO, the most appropriate timing for tissue cryopreservation and autologous re-transplantation is still debated (Mamsen *et al.*, 2020; Missontsa *et al.*, 2024). In some studies, spontaneous pubertal development and menstrual cyclicity have been reported (Donnez *et al.*, 2007, 2010; Missontsa *et al.*, 2024). Few births after ovarian tissue transplantation have been described in patients affected by β-TM and SCA (Roux *et al.*, 2010; Revel *et al.*, 2011; Demeestere *et al.*, 2015; Matthews *et al.*, 2018; Missontsa *et al.*, 2024).

In both sexes, early referral to a specialized team in reproductive medicine, including an andrologist or a gynaecologist, is essential to ensure appropriate counselling and timely intervention.

### Pre-conceptional evaluation in couples

As for standard procedure in couple infertility, both partners should undergo thorough andrological and gynaecological evaluations, with special attention to endocrine status and systemic complications in the affected individual.

Genetic testing aimed at identifying hereditary traits underlying the IO condition is essential to guide decision-making and actuate preventive measures (Leung and Lao, 2012). Indeed, in selected cases, carrier screening in the unaffected partner is also advised. For instance, when a pathogenic variant is identified in one or both partners, genetic counselling should be offered to evaluate the implications and to consider preimplantation genetic testing for monogenic conditions.

In females with IO, additional investigations should address the systemic iron burden and its impact not only on reproductive potential but also on overall health, due to the substantial physiological demands of pregnancy. Quantitative T2* MRI is essential for assessing iron deposition in the liver, myocardium, and pituitary gland. Myocardial T2* values <20 ms are associated with a significantly increased risk of cardiac dysfunction (Anderson *et al.*, 2001; Au *et al.*, 2008) and warrant preconception counselling and, when appropriate, intensification of ICT (Au *et al.*, 2008; Farmaki *et al.*, 2010; Poggi *et al.*, 2016). ICT is generally discontinued ∼3 months before conception, with deferoxamine discontinued immediately prior, and can be resumed immediately after delivery (Bajoria and Chatterjee, 2011; Cassinerio *et al.*, 2017).

As discussed in the ‘Hypogonadism’ section, both iron toxicity and estrogen deficiency may impair BMD. In females planning a pregnancy, the evaluation of bone health becomes even more relevant, since gestation and lactation are associated with increased calcium mobilization and accelerated bone turnover, potentially exacerbating pre-existing bone fragility and increasing fracture risk (Kovacs, 2016; Orhadje *et al.*, 2025).

#### Male partner evaluation

The evaluation of the male partner should start with a detailed clinical history and a standard physical and andrological examination to identify risk factors for infertility. For instance, symptoms of hypogonadism and systemic complications of the underlying IO disorder should be identified, as well as acquired causes of infertility, e.g. varicocele, cryptorchidism, previous trauma, orchitis, or infections of the urogenital tract. Serum inhibin B (INHB) levels, a direct marker of Sertoli cell function and an indirect marker of spermatogenesis, are known to be associated with sperm concentration both in men with normal (Jensen *et al.*, 1997b) and impaired spermatogenesis (Klingmuller and Haidl, 1997). As such, INHB might predict spermatogenic function in a screening setting before a semen analysis is carried out (Makanji *et al.*, 2014). Nonetheless, no studies have so far assessed its reliability in the context of IO disorders. Finally, semen analysis, conducted following the sixth edition of the ‘WHO laboratory manual for the examination and processing of human semen’ (World Health Organization, 2021), is central to this assessment. The analysis of at least two semen collections is recommended to account for intra-individual variability. The routine semen analysis will provide information on standard parameters, such as semen volume, sperm concentration and total number, motility, morphology, and vitality, if needed. Details on endocrine diagnostic evaluation have already been described in the ‘Hypogonadism’ section and are summarized in Fig. 1.

Scrotal colour-Doppler Ultrasound (CDUS) is the gold standard for evaluating testicular morphology (Pozza *et al.*, 2020; Lotti *et al.*, 2022; Pozza *et al.*, 2023) and should be performed when clinical findings warrant further assessment. CDUS represents an important diagnostic tool providing information on parameters such as testicular volume, echotexture, and vascularization, among others. Although studies focusing on testicular appearance in the context of IO are lacking, this imaging modality can identify early structural or functional abnormalities, such as reduced testicular perfusion (Carlomagno *et al.*, 2022), fibrosis (represented by markedly inhomogeneous echotexture (Harris *et al.*, 2000; Loberant *et al.*, 2010)) or microlithiasis (Pozza *et al.*, 2020), and together with hormonal analysis can help distinguish primary testicular damage from secondary causes of spermatogenic failure.

Given that genetic causes of azoospermia or OAT may coexist in patients with chronic conditions, standard genetic investigations, including karyotype analysis and Y-chromosome microdeletion testing, should be performed in accordance with established literature for the evaluation of male infertility (Colpi *et al.*, 2018; Krausz and Riera-Escamilla, 2018). These genetic tests are especially indicated when pre-existing OAT or azoospermia are documented. Karyotype analysis should be performed in men with azoospermia or oligozoospermia <10 million spermatozoa/ml (Jungwirth *et al.*, 2012). Y-chromosome microdeletion screening is requested in case of <5 million spermatozoa/ml (Krausz *et al.*, 2024). The identification of a genetic cause for spermatogenic failure has important implications for the clinical management of the affected individual.

#### Female partner evaluation

The diagnostic evaluation of the patient with gonadal impairment has been partially anticipated in the ‘Hypogonadism’ section. It should include the assessment of AMH levels, preferably measured using a validated and reliable assay, as a biomarker of ovarian reserve. Serum INHB levels, measured during the early follicular phase, have also been used to estimate ovarian reserve and predict ART outcomes (Makanji *et al.*, 2014); however, its use is limited compared to AMH (Anderson *et al.*, 2012), and therefore this measurement is not carried out routinely. Lastly, serum progesterone levels could be measured in spontaneously menstruating women to assess whether ovulation has occurred. The assay should be carried out in the luteal phase, specifically ∼7 days before the expected date of menstruation, and a value >3 ng/ml is typically considered indicative of ovulation (Prior *et al.*, 2015).

In parallel, transvaginal pelvic ultrasonography should be performed to assess both ovarian volume and AFC, which, when integrated with AMH, provide a comprehensive estimation of functional ovarian reserve (Singer *et al.*, 2010, 2011). In addition, this imaging modality allows the detailed assessment of endometrial thickness, an important parameter in evaluating peripheral estrogen action and endometrial receptivity and uterine development. The latter is particularly relevant in this clinical setting, as inappropriate pubertal induction or exposure to pelvic or total body irradiation (TBI) often results in uterine hypoplasia or scarring, further complicating fertility potential (Karabulut *et al.*, 2010; Federici *et al.*, 2022). As part of standard clinical practice for patients undergoing ART, it is advised to assess thyroid function, including thyroid stimulating hormone (TSH) and free T4 levels, and, if indicated, thyroid autoantibodies.

### Treatment options for infertility

Natural conception remains a realistic option in many cases, particularly in milder forms of IO, which may be compatible with valid spermatogenesis and ovulatory cycles. Alternatively, as infertility in both males and females with IO disorders frequently originates from pituitary dysfunction, resulting in hypogonadotropic hypogonadism, gonadotropin stimulation represents a rational and effective therapeutic approach, provided that no primary gonadal damage is present. By directly compensating for inefficient or absent gonadotropin secretion, this strategy can help restore steroidogenic and reproductive functions and enable spermatogenesis or oocyte development and ovulation.

#### Male infertility: gonadotropin therapy

Therapy with (recombinant or highly purified) FSH in men, particularly those with OAT or azoospermia, and normal or low FSH levels, may restore spermatogenesis, although data remain scarce in this specific population. In men with hypogonadotropic hypogonadism, treatment should also include hCG to stimulate endogenous testosterone production. Available evidence, although limited, suggests that combined gonadotropin therapy can induce spermatogenesis in a subset of men with hypogonadotropic hypogonadism. Successful induction has been reported in small cohorts and case reports, including in 6 out of 14 patients (Bajoria and Chatterjee, 2011), 3 out of 4 (De Sanctis *et al.*, 1988), and 2 out of 5 with β-TM (De Sanctis *et al.*, 2019a). Since many patients with IO may have initiated TRT prior to attempting conception, combined gonadotropin therapy should be preceded by an adequate wash-out period of exogenous testosterone. Gonadotropin therapy is typically administered for at least 6–12 months. Semen analysis should be repeated every 3 months to assess treatment efficacy, and cryopreservation is encouraged following successful stimulation.

#### Female infertility: ovulation induction and ovarian stimulation

In women with ovulatory dysfunction, induction of ovulation can be attempted using letrozole or clomiphene citrate, provided that the ovarian reserve and uterine condition are adequate (Conforti *et al.*, 2025). If first-line approaches fail, follicular development and ovulation can be effectively induced in women with amenorrhoea using gonadotropin therapy (hMG/FSH combined with hCG). In women with primary or secondary amenorrhoea due to β-TM, ovulation was achieved in ∼75% of cases, with dosage adjustments guided by transvaginal ultrasound monitoring (Danesi *et al.*, 1994; Castaldi and Cobellis, 2016). Additionally, increases in both ovarian and uterine volume have been reported following stimulation in women with primary amenorrhoea (Danesi *et al.*, 1994). Several protocols are available for ovarian stimulation and should be selected based on patient characteristics, including age, ovarian reserve, cardiometabolic status, and iron-related comorbidities. Conventional protocols employing recombinant FSH or hMG injections, often in combination with a GnRH antagonist to prevent premature LH surge, are commonly used due to their flexibility and safety profile (Shah *et al.*, 2024; Conforti *et al.*, 2025).

#### Conception through ART

Thanks to advances in reproductive medicine, ARTs represent valuable symptomatic treatment options for infertile or subfertile patients of both sexes affected by IO disorders. ART referral should be considered in males with normal sperm parameters after 12 months without conception in the presence of a female partner with no risk factors for infertility. Semen cryopreservation is strongly recommended following successful stimulation, even in cases of low sperm counts or when sperm parameters are suboptimal (Cappellini *et al.*, 2021). Indeed, IVF/ICSI can be used in OAT patients with and without previous hormonal treatment.

In females, ART is advisable after six ovulatory cycles without obtaining a pregnancy, despite timed intercourse and a male partner with normal sperm parameters (Shah *et al.*, 2024). Early referral is warranted in older women or those with additional risk factors affecting ovarian reserve or tubal function. Success rates of such interventions are influenced by several factors, including age, degree of IO, and the extent of damage at the germinal epithelium (Mensi *et al.*, 2019). Interestingly, higher iron concentrations in the follicular fluid of women undergoing IVF techniques are also associated with worse embryological outcomes (Gonzalez-Martin *et al.*, 2024).

Pregnancy outcomes in women with IO disorders have significantly improved over time, thanks to optimized transfusion protocols, ICT, and ART. In our review of the available literature (Table 6), covering 3536 pregnancies in 3131 women, we found that ART, including ovarian stimulation, IUI, IVF, and/or ICSI, was employed in 19.4% of pregnancies. To better reflect the actual prevalence of advanced reproductive techniques, we excluded ovulation induction with clomiphene or letrozole alone from this estimate. This choice was made in light of the heterogeneity and limited granularity of available data and aims to more accurately capture the subset of patients requiring structured intervention in reproductive medicine, beyond pharmacologically induced ovulation.

### Pregnancy risks and outcomes

Pregnancy in women with IO disorders presents specific challenges. According to our pooled analysis (Table 6), the rate of miscarriage was 11.2%, in line with that of the general population (Quenby *et al.*, 2021), while premature delivery occurred in 9.8% of pregnancies, a comparable rate to global estimates (Ohuma *et al.*, 2023). Caesarean section was performed in 29.7% of cases, compared to a worldwide rate of ∼21% (Betran *et al.*, 2021), reflecting both obstetric indications and precautionary approaches to minimize risks related to maternal or foetal outcomes. The mean gestational age was 37.1 ± 3.1 weeks, and the average birth weight was 2.64 ± 0.68 kg, consistently lower than the general population average (Bonanni *et al.*, 2024), likely due to maternal anaemia and disease-related complications.

These outcomes are linked to the pathophysiological effects of IO during pregnancy. Physiological changes during gestation typically include a decline in haemoglobin levels and a rise in median ferritin levels, reflecting increased hepatic iron burden and altered iron metabolism (Cassinerio *et al.*, 2017). These changes contribute to significant obstetric risks, including spontaneous miscarriage, pre-eclampsia, and placental complications (Cassinerio *et al.*, 2017). Women with a history of pelvic irradiation or TBI face additional risks due to uterine damage, leading to preterm delivery, low birthweight, or miscarriage. As reported in the ‘Pre-conceptional evaluation in couples’ section above, pregnancy should be planned during periods of stable disease and optimal management of IO and its systemic complications to reduce the risk of maternal and foetal complications and improve overall pregnancy outcomes.

However, even in the absence of the above-mentioned known risk factors, pregnancy-related complications remain frequent. According to a 2022 French national registry study on 75 pregnancies from 37 women with β-TM and β-TI, the overall caesarean section rate was 58.5%, reflecting precautionary strategies to minimize risks for both the mother and the foetus (Virot *et al.*, 2022). This figure, higher than the pooled figure reported above across all IO disorders, likely reflects differences in diagnoses, clinical practice, and nation-specific protocols. In fact, although guidelines state that thalassaemia alone is not an indication for a caesarean section (Taher *et al.*, 2020; Shah *et al.*, 2024), it is often recommended as mode of delivery due to concerns related to cardiac function and liver dysfunction (Tuck, 2005; Origa *et al.*, 2010). Obstetrical complications, such as pregnancy-induced hypertension, pre-eclampsia, gestational diabetes, and haemorrhages, at birth are also frequent, contributing to the elevated risk profile in this patient population (Virot *et al.*, 2022).

In addition to obstetric risks, pregnancy may exacerbate systemic complications such as heart failure, endocrine dysfunctions (including diabetes, hypoparathyroidism, and osteoporosis (Leung and Lao, 2012; Poggi *et al.*, 2016)), and thrombotic events, particularly in splenectomized individuals, who are predisposed to hypercoagulability due to the combined effects of splenectomy and chronic anaemia (Bajoria and Chatterjee, 2009). Prophylactic anticoagulation is therefore recommended in this subgroup (Shah *et al.*, 2024). Though maternal deaths are rare, they have been reported, particularly due to postpartum cardiac complications (Tuck, 2005).

Despite these risks, most pregnancies can result in live births when properly supported by appropriate planning, close monitoring, and multidisciplinary care. Notably, advances in ICT, optimized transfusion regimens, and improved obstetric management have contributed to markedly better obstetric and neonatal outcomes in recent years. In the postpartum period, a prompt resumption of ICT is required to address the increased iron burden accumulated during gestation (Cassinerio *et al.*, 2017).

## Impact of ICT and future therapeutic perspectives

ICT plays a pivotal role in mitigating the systemic consequences of IO, including its detrimental effects on gonadal function. However, the efficacy of different chelation agents in preserving reproductive health varies significantly, influencing the prevalence and severity of hypogonadism in patients with TDT.

In a large cohort of 713 (388 female and 325 male) patients undergoing ICT, the prevalence of hypogonadism differed depending on the chelation agent: 24.5% with deferasirox, 16.8% with deferoxamine, 6.5% with deferiprone, and 52.5% in those receiving combination regimens (Bordbar *et al.*, 2019). Splenectomy was associated with a nearly 2-fold increase in the risk of hypogonadism, possibly due to reduced total iron-binding capacity and increased serum ferritin levels and tissue iron deposition. Alternatively, patients undergoing a more intensive ICT may have had a higher baseline iron burden, often accompanied by severe splenomegaly, leading to worse pituitary siderosis and subsequent endocrine dysfunction.

Historically, the timing of ICT initiation was a major determinant of gonadal outcomes in patients with TDT. In an early cohort of 40 female patients with β-TM, initiation of deferoxamine before the age of 10 years was associated with normal pubertal development and preserved gonadotropin responses, whereas delayed initiation was linked to primary amenorrhoea and hypogonadotropic hypogonadism (Bronspiegel-Weintrob *et al.*, 1990). These findings informed current guidelines recommendations advocating for early initiation of ICT in transfused children (Shah *et al.*, 2022; Shah *et al.*, 2023; Taher *et al.*, 2025).

The type of chelation therapy also influences endocrine outcomes. In a multicentre retrospective Italian study of 86 patients with β-TM (57 females and 29 males), treated with once-daily deferasirox for a median of 6.5 years, new-onset hypogonadism was observed in only five patients (Casale *et al.*, 2014). Similarly, a 5-year longitudinal study in 165 adult patients with β-TM found a slightly lower prevalence of hypogonadism in those treated with deferasirox compared to other agents (deferoxamine, deferiprone, or combinations) (Poggi *et al.*, 2016).

Combined intensive ICT regimens may provide additional benefits in reversing or mitigating gonadal dysfunction. In a cohort of 26 hypogonadal women (19 with primary or secondary amenorrhoea) treated with a combination of deferoxamine and deferiprone for 5–7 years, significant hormonal improvements were observed, including increases in basal and GnRH-stimulated gonadotropins and estradiol levels. Remarkably, six women achieved pregnancy: two spontaneously and four with the use of ART (Farmaki *et al.*, 2010). Furthermore, it has also been proposed that effective ICT may improve ovarian function indirectly by enhancing insulin sensitivity as observed in some studies on diabetic patients with IO undergoing phlebotomy (Dymock *et al.*, 1972; Niederau *et al.*, 1996; Stechemesser *et al.*, 2017).

As described, although ICT represents the cornerstone of treatment for systemic IO, its ability to reverse established reproductive dysfunction is limited. This apparent dissociation between effective systemic iron removal and incomplete recovery of gonadal function suggests that factors beyond circulating iron concentrations play a critical role. Available evidence suggests that the efficacy of chelators is constrained by restricted penetration into specific tissue compartments. Specifically, the limited ability of ICT to penetrate protected tissue compartments, exemplified by its poor blood–brain barrier permeability (Finkenstedt *et al.*, 2010), illustrates a broader challenge in achieving effective chelation at the tissue level, which may extend to other specialized barriers, including the blood–testis barrier (Hau *et al.*, 2023; Luaces *et al.*, 2023). To address these limitations, experimental strategies aimed at enhancing tissue-specific delivery have been explored. In preclinical models, nanoparticle-based formulations combined with iron chelators have been shown to improve tissue penetration and reduce oxidative stress and DNA damage more effectively than chelators alone (Adel *et al.*, 2021; Onrubia-Marquez *et al.*, 2025; Zhao *et al.*, 2025).

While these approaches remain confined to experimental settings, they provide proof-of-concept that overcoming pharmacokinetic and tissue barriers may enhance the therapeutic modulation of iron-induced ferroptosis.

Importantly, these pharmacokinetic and anatomical constraints alone may not fully explain the limited reversibility of reproductive dysfunction. Beyond iron removal, increasing attention has been directed toward the downstream molecular mechanisms through which iron excess induces cellular damage in reproductive tissues, since preclinical studies indicate that iron chelation may only partially counteract oxidative damage once ferroptotic pathways are activated (Zhang *et al.*, 2024). Experimental modulation of ferroptosis has shown promising protective effects in reproductive models. Direct inhibition of lipid peroxidation using small-molecule ferroptosis inhibitors, such as ferrostatin-1 and liproxstatin-1, attenuates ferroptotic cell death and preserves cellular viability in iron- and oxidative stress-exposed tissues (Dixon *et al.*, 2012; Friedmann Angeli *et al.*, 2014; Zhang *et al.*, 2024). In germ cell systems, suppression of lipid peroxidation and modulation of key regulators of membrane polyunsaturated fatty acid composition, including ACSL4, reduce susceptibility to ferroptotic injury (Bromfield *et al.*, 2019; Moretti *et al.*, 2024). Reinforcement of endogenous antioxidant defences along the System Xc^−^/glutathione/GPX4 axis similarly limits lipid peroxide accumulation and mitigates ferroptosis-mediated cellular damage (Stockwell *et al.*, 2017; Zhang *et al.*, 2024).

At present, these approaches remain confined to experimental settings, and no clinical trials have evaluated ferroptosis-targeting interventions in patients with IO-associated reproductive dysfunction. Nevertheless, while clinical data are lacking, findings from experimental models and human reproductive tissues indicate that ferroptosis-related alterations can be detected in tissues exposed to iron excess, including testicular tissue, semen, and the placenta (Huo *et al.*, 2008; Liao *et al.*, 2023; Moretti *et al.*, 2024; Wang *et al.*, 2025). These observations support the biological plausibility of ferroptosis as a contributor to iron-mediated reproductive damage. Future therapeutic development should prioritize the validation of ferroptosis biomarkers in well-characterized cohorts with IO, the integration of tissue-specific measures of iron burden and lipid peroxidation, and the exploration of combined strategies in which conventional ICT is complemented by targeted modulation of ferroptotic pathways.

Taken together, these findings underscore the importance of tailored ICT strategies based on the patient’s clinical profile, iron burden, and stage of reproductive involvement. Early initiation and appropriate selection of chelation regimens remain critical to minimize the risk of hypogonadism and preserve fertility potential in this high-risk population. At the same time, the emerging recognition of tissue-specific limitations and ferroptosis-driven damage highlights that ICT should primarily be viewed as a preventive intervention rather than a fully restorative one. Future advances will therefore likely depend on integrated approaches in which timely and personalized chelation is complemented by targeted strategies aimed at protecting reproductive tissues from iron-induced cellular injury.

## Conclusions

In interpreting the available evidence, several overarching considerations deserve explicit acknowledgement. Much of the current knowledge on reproductive and sexual dysfunction in IO disorders emerges from heterogeneous clinical contexts, spanning different aetiologies, degrees and timing of iron accumulation, and evolving standards of chelation therapy. This heterogeneity, rather than representing a simple methodological limitation, reflects the biological and clinical complexity of iron toxicity across the lifespan and across organs with different vulnerability windows, particularly during pubertal development and early adulthood. In addition, while mechanistic pathways such as ferroptosis provide a unifying conceptual framework linking iron accumulation to gonadal damage, validation in humans remains fragmentary and largely indirect, highlighting the gap between experimental models and clinically accessible tissue-level endpoints. Sexual health outcomes, especially in women, remain strikingly under-investigated, with a near-complete absence of studies employing validated psychometric tools, despite the high prevalence of hypogonadism and its psychosocial burden. Finally, although ICT has transformed survival and reduced systemic complications, its impact on reproductive dysfunction appears predominantly preventive rather than restorative, underscoring the importance of early intervention and the need to move beyond circulating iron metrics toward tissue-specific and pathway-oriented markers of damage.

IO disorders, arising from genetic abnormalities in iron metabolism or secondary to conditions requiring recurrent blood transfusions, lead to progressive iron accumulation in target organs, culminating in multiorgan damage.

The endocrine system is particularly vulnerable to iron toxicity, with hypogonadism emerging as the most frequent complication in both sexes. Iron deposition primarily affects the HPG axis, leading to hypogonadotropic hypogonadism or, less commonly, mixed hypogonadism that involves both central and gonadal levels. The resulting hormonal deficiencies manifest through different symptoms in both sexes, such as delayed or arrested pubertal development, reduced libido, sexual dysfunction, infertility (primary or secondary amenorrhoea in women, azoospermia or OAT in men), and increased risk for metabolic syndrome, cardiovascular disease, and osteoporosis.

Effective management of IO disorders requires the timely initiation and optimization of ICT regimens to mitigate iron toxicity and its systemic consequences. ERT should be tailored to address sex hormone deficiencies, alleviate sexual dysfunction, and prevent long-term complications such as osteoporosis and cardiovascular disease. For couples seeking to conceive, a standard evaluation of the gonadal function and general health issues are mandatory to optimize fertility outcomes, either improving the chance for natural or ART pregnancies. Pre-conceptional genetic counselling should also be integrated into the multidisciplinary evaluation. In both sexes, preservation of fertility through gamete cryopreservation is a feasible option. Individualized treatment plans, leveraging the combined expertize of endocrinologists, haematologists, andrologists, gynaecologists, and reproductive medicine experts, play a pivotal role in improving both fertility and overall health status of the patient. Personalized treatment options contribute to successful conception, prevention of disease progression, and improvement of quality of life.

Despite significant advancements, notable knowledge gaps remain. Key areas requiring further investigation include the use of advanced imaging techniques, such as testicular CDUS and pituitary MRI, in the detection of iron-induced organ damage, the exact prevalence of mixed forms of hypogonadism and of impaired spermatogenesis, the long-term safety of TRT/ERT, and ART success rates and fertility outcomes in this population. Moreover, the extent to which sex-related biological differences affect the progression of gonadal dysfunction, the response to iron chelation, and the efficacy of hormonal and fertility treatments remains poorly understood. Future studies should therefore prioritize sex-disaggregated data and longitudinal designs to delineate these differences and tailor interventions accordingly.

Ultimately, refining diagnostic and therapeutic protocols and embedding reproductive health in the routine management of IO disorders will be key to improving not only fertility outcomes, but also long-term health and quality of life for both male and female patients.

## Data Availability

All data are incorporated into the article and its accompanying materials.

## Abbreviations list

β-TM: β-thalassaemia major
β-TI: β-thalassaemia intermedia
AFC: antral follicle count
BMD: bone mineral density
CDUS: colour-Doppler ultrasound
DXA: dual energy X-ray absorptiometry
E2: estradiol
EAA: European Academy of Andrology
ED: erectile dysfunction
ERT: estrogen replacement therapy
Fe2^+^: ferrous iron
Fe3^+^: ferric iron
GHD: growth hormone deficiency
GPX4: glutathione peroxidase 4
HH: hereditary haemochromatosis
HPG: hypothalamic–pituitary–gonadal
ICT: iron chelation therapy
INHB: inhibin B
IO: iron overload
LTCC: L-type Ca^2+^ channels
MRI: magnetic resonance imaging
(N)-TDT: (non-)transfusion-dependent thalassaemia(s)
OAT: oligoasthenoteratozoospermia
PCOS: polycystic ovary syndrome
PUFA: polyunsaturated fatty acids
ROS: reactive oxygen species
SCA: sickle cell anaemia
SHBG: sex hormone-binding globulin
(T/cf)T: (total/calculated free) testosterone
TBI: total body irradiation
TfR: transferrin receptor
TRT: testosterone replacement therapy
